# Environmental Phenol and Paraben Exposure Risks and Their Potential Influence on the Gene Expression Involved in the Prognosis of Prostate Cancer

**DOI:** 10.3390/ijms23073679

**Published:** 2022-03-27

**Authors:** Diaaidden Alwadi, Quentin Felty, Deodutta Roy, Changwon Yoo, Alok Deoraj

**Affiliations:** 1 Department of Environmental Health Sciences, Florida International University, Miami, FL 33199, USA; dalwa002@fiu.edu (D.A.); feltyq@fiu.edu (Q.F.); droy@fiu.edu (D.R.); 2 Biostatistics Department, Florida International University, Miami, FL 33199, USA; cyoo@fiu.edu

**Keywords:** environmental phenols, paraben, prostate cancer, gene ontology, NHANES, protein–protein interaction

## Abstract

Prostate cancer (PCa) is one of the leading malignant tumors in US men. The lack of understanding of the molecular pathology on the risk of food supply chain exposures of environmental phenol (EP) and paraben (PB) chemicals limits the prevention, diagnosis, and treatment options. This research aims to utilize a risk assessment approach to demonstrate the association of EP and PB exposures detected in the urine samples along with PCa in US men (NHANES data 2005–2015). Further, we employ integrated bioinformatics to examine how EP and PB exposure influences the molecular pathways associated with the progression of PCa. The odds ratio, multiple regression model, and Pearson coefficients were used to evaluate goodness-of-fit analyses. The results demonstrated associations of EPs, PBs, and their metabolites, qualitative and quantitative variables, with PCa. The genes responsive to EP and PB exposures were identified using the Comparative Toxicogenomic Database (CTD). DAVID.6.8, GO, and KEGG enrichment analyses were used to delineate their roles in prostate carcinogenesis. The plug-in CytoHubba and MCODE completed identification of the hub genes in Cytoscape software for their roles in the PCa prognosis. It was then validated by using the UALCAN database by evaluating the expression levels and predictive values of the identified hub genes in prostate cancer prognosis using TCGA data. We demonstrate a significant association of higher levels of EPs and PBs in the urine samples, categorical and numerical confounders, with self-reported PCa cases. The higher expression levels of the hub genes (BUB1B, TOP2A, UBE2C, RRM2, and CENPF) in the aggressive stages (Gleason score > 8) of PCa tissues indicate their potential role(s) in the carcinogenic pathways. Our results present an innovative approach to extrapolate and validate hub genes responsive to the EPs and PBs, which may contribute to the severity of the disease prognosis, especially in the older population of US men.

## 1. Introduction

The United States Food and Drug Administration (FDA) and the Environmental Protection Agency (EPA) estimate more than 10,000 chemicals are added directly or indirectly to the human food supply chain [1,2]. These occurrences have led to investigations involving the association of the toxic effects of these chemicals on cancer and other chronic diseases [3,4]. The increasing concern regarding human exposure to endocrine disrupting chemicals (EDC) and their toxic effects on cancer and chronic diseases is evident by the sheer number of scientific publications in the last two decades (Figures S1 and S2). Concomitantly, endocrine-disrupting properties of environmental phenols (EP) and paraben (PB) exposures to humans have also been suggested in laboratory research and animal models [5].

Prostate cancer (PCa) is the most prevalent hormone-dependent cancer and the second leading cause of cancer deaths in US men. In 2021, there were an estimated 248,530 new cases (and 34,130 deaths) in the US men [6], an approximate 56,600 increase from 191,930 cases reported in 2020 [7]. The highest number of PCa cases is diagnosed in men >65 years and older and in non-Hispanic Blacks with 60% [6,8]. Studies indicate that heritability and germline mutations of BRCA1 and BRCA2 may play a significant role in developing PCa, contributing to approximately 57% of the cases [7,9,10]. Studies also show that EDCs exposure in early life increases susceptibility to prostate cancer in rodent models and the association of elevated urinary concentration with PCa in humans [11,12]. There is widespread use of products containing EPs and PBs with endocrine disrupting properties; therefore, their exposure is prevalent. The exposure can occur through ingestion, absorption through the skin, or inhalation. Women and children tend to be more exposed than men given the types of products in which these chemicals are used. We selected EPs (including BPA) and PBs for our investigation because they are listed in the EPA’s existing Toxic Substances Control Act (TSCA) to ascertain whether they present an extreme risk to human health and environmental contamination under the circumstances of their use [13,14]. In addition, The National Health and Nutrition Examination Survey (NHANES) datasets have been intensively used to investigate the association between chemicals, metabolites, and different chronic diseases and cancers [15,16,17,18,19,20,21]. Assuming the potentially toxic effects, several studies, as well as samples collected by NHANES, monitored specific EP and PB and their metabolite concentrations in the serum [22,23], urine [22,24], and breast milk [22,25].

Several EPs were considered in this study because of their suggested endocrine disrupting roles in different cancers due to their estrogenic or androgenic action and a possible connection with PCa [26,27]. BPA [(CH_3_)_2_ C(C_6_H_4_OH)_2_)] is an organic and synthetic compound utilized in the production of polycarbonate plastics, epoxy, and phenolic resins [28]. Ingestion of BPA is recognized as the principal route of human exposure (>90%), which is estimated at around 3 to 11 μg /kg/BW/daily [28,29], compared with dust at 0.03 to 0.3 μg /daily (<5%) [30], thermal paper at 0.5 to 1.3 μg /daily (<5%) [31], and dental surgery at 0.2 μg /daily μg /daily (<5%) [32]. BPA mimics weak estradiol and binds to estrogen receptors influencing cell proliferation and growth modifications to contribute to the spreading and progressions of ovarian [33,34], cervical [35], breast [36], and prostate [37] cancers. The induction of carcinogenesis by BPA in animal models has also been studied [38,39,40,41] to show the incidence of prostate neoplasia and neoplasms. Benzophenone-3 [C_14_H_12_O_3_] is an organic solvent found in products such as food packaging, lotion, and sunscreen with a half-life between 5 and 10 h [42]; Triclosan [C_12_H_7_C_l3_O_2_] is used as a germ-killing agent with a half-life of nearly 10 to 15 h [43]; and 4-tert-Octyl phenol [C_14_H_22_O] is an alkylphenol utilized to produce anionic surfactants. The PBs are organic and estrogenic chemicals with suggested endocrine disrupting roles with a half-life of fewer than 24 h and are broadly used in food and beverage processing, pharmaceutical products, and personal care products [5,23,44]. The most utilized PBs are Methylparaben [C_8_H_8_O_3_), Ethylparaben [C_9_H_10_O_3_], Propylparaben [C_10_H_12_O_3_], and Butylparaben [C_11_H_14_O_3_]. The chemical structures of all the EPs and PBs investigated in this study are presented in Figure S3. PB compounds show higher estrogenic activity in a stably transfected transcriptional activation assay (STTA) compared to the cell proliferation assay [45] and are detectable in human urine samples [45]. These EPs and PBs were also continuously measured in the urine samples during the five cycles of NHANES data (2005–2015).

Epidemiological and statistical association studies of EDCs or their metabolites with various human cancers lack information on the underlying causality of the exposures. The PSA is a prototypic androgen receptor that is utilized as one of the early diagnostic biomarkers for scanning PCa. However, there remain uncertainties in screening for PCa by PSA levels, which are influenced by various stimuli, ranging from inflammation to sexual activity, leading to overdiagnosis and overtreatment of PCa [46,47]. Some studies have determined numerous differentially expressed genes (DEGs) and biomarkers through microarrays analysis in the various biological processes and pathways of PCa progression [46,48,49,50,51]. The lack of understanding of the molecular pathology on the risk of environmental phenol (EP) and paraben (PB) chemical exposures further limits the prevention, diagnosis, and treatment options. Therefore, to strengthen the association of the EPs and PBs with PCa, it is essential to investigate the molecular pathogenesis of prostate carcinogenesis. The genes responsive to EP and PB exposures were analyzed using curated databases of the Comparative Toxicogenomic Database (CTD) and DAVID.6.8, and GO and KEGG enrichment analyses to delineate the roles of DEG in prostate carcinogenesis.

In this study, we: (1) applied a risk assessment approach to investigate the association of PCa cases with the levels of EPs and PBs in the urine samples of US men and 17-epidemiological covariates in the US men by comprehensibly utilizing the NHANES (2005–2015) data, and (2) investigated various influenced pathways of EPs and PBs in the etiology of PCa to discover hub genes for the development of robust diagnostics or therapeutic molecular targets for US men.

## 2. Results

### 2.1. Descriptive Statistics for Male Population—NHANES Data (2005–2015)

There were 4592 men of ≥20 years of age included in this study for which EPs and PBs NHANES data were publicly available for five cycles between the years 2005 and 2015. These individuals responded to the medical condition questionnaire. Among the 4592 male participants, 4440 (96.7%) did not report PCa incidence, while 152 (3.7%) reported PCa diagnosis (Table 1) on their medical questionnaire. There were nine (0.2%), sixty-one (1.4%), and eighty-one (1.7%) reported cases of PCa in the age groups of 20–49, 50–69, and ≥70, respectively. There were more men of non-Hispanic White ethnicity among the non-cases (46.1%) and in the PCa cases (1.7%) compared with the non-Hispanic Blacks and others in the selected population for this study. The BMI records in the study population were categorized as normal (<25 kg/m^2^), overweight (25 to 30 kg/m^2^), and obese (≥30) for 39.2%, 30.6%, and 30.2%, respectively, of the selected population. The annual family income was reported to be ≤$24,999 (28.1%) and $25,000 to $54,999 (32.2%) in the study population, including cases and non-cases, and 54.9% of the population had ≤12th grade of education only. The record showed that 68.9% of the population reported yes to alcohol consumption, 74.7% were born in the US, 55.5% reported no physical activity, and 61.8% stated that they did not eat frozen food. Approximately 50% of the respondents were smokers. To consider the physiological conditions of the included subjects in this study, we recorded that 96.7% had no incidence of liver disease, and 96.6% had no incidence of kidney disease (Table 1). When the population filled out the questionnaire, the mean bodyweight of the population with reported cancer cases was 86 Kg, and their mean age was 69 years and over. The mean bodyweight of the subjects without PCa was 76 Kg, and their mean age was found to be 50 years (Table 2). The mean serum concentrations of total cholesterol, LDL, and triglycerides were measured to be higher in the men with PCa in groups (228, 199, 246 mg/dL, respectively) than the men who did not report PCa diagnosis (179, 106, 98 mg/dL, respectively), as shown in Table 2.

### 2.2. EP and PB Levels in the Urine Samples of PCa Subjects

**Figure 1:**
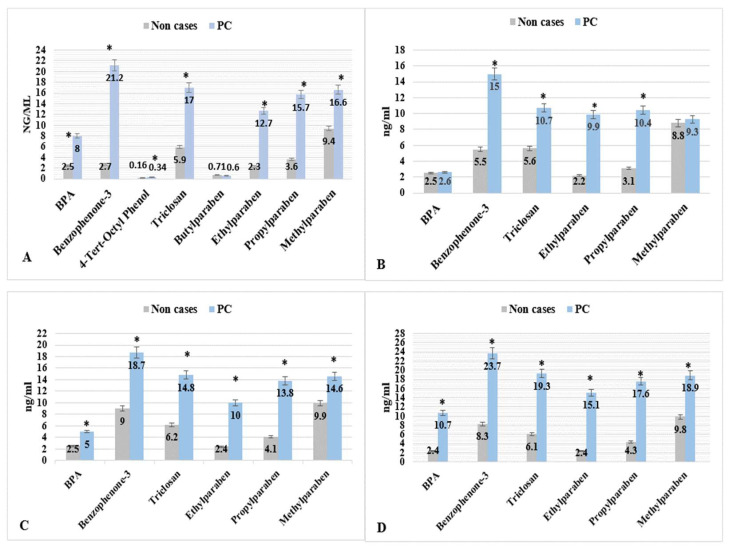
EPs & PBs levels (mean, ng/mL) by PCa for men with concentrations ≥ LOD; NHANES 2005–2015. * *p* < 0.05. The four parts are (A) men with age ≥ 20 years, (B) men with age between 20–49 years, (C) men with age between 50–69 years, and (D) men with age ≥ 70 years).

Table 3 shows the statistical summary of EPs and PBs by PCa status. The means of EPs and PBs levels were significantly higher for seven metabolites, except Propylparaben, in the men who reported PCa diagnosis than males who reported no PCa diagnosis. The mean levels of Eps, such as BPA, Benzophenone-3, 4-Tert-Octyl Phenol, and Triclosan, were significantly higher in PCa cases compared with non-cases (8.0, 2.5 ng/mL), (21.2, 7.1 ng/mL), (0.34, 0.16 ng/mL), and (17, 9.5 ng/mL), respectively. The mean levels of the three PBs, Ethylparaben, Methylparaben, and Butylparaben, were significantly higher in PCa cases compared with non-cases (12.7, 2.3 ng/mL), (16.6, 9.4 ng/mL), and (15.7, 3.6 ng/mL), respectively (Table 3 and Figure 1). The maximum urine levels reported in the PCa cases for EPs and PBs were Benzophenone-3 (29.0 ng/mL) and Methylparaben (25.0 ng/mL).

### 2.3. Association of Metabolite Levels, Categorical Variables, and PCa

Tables S1–S6 show the arithmetic means of the urine levels of six EPs and PBs for PCa cases and non-cases for chosen variables in the study population. The arithmetic means of the six EP and PB metabolites were significantly higher in PCa cases than in non-cases, particularly in groups 50–69 and ≥70years old. Table 4 presents the age, weight, and lipid profile levels (mg/dL) in the male populations. The arithmetic means age (69 years old) for the PCa cases was significantly higher compared with the mean age (50 years old) for non-cases. The arithmetic means weight (86 Kg) for the PCa cases was significantly higher than the mean age (76 Kg) for non-cases. For the lipid profiles, the total serum cholesterol (228 mg/dL), serum LDL (199 mg/dL), and serum triglycerides (247 mg/dL) for the PCa cases were significantly higher compared with the total serum cholesterol (179 mg/dL), serum LDL (106 mg/dL), and serum triglycerides (98 mg/dL) for non-cases. However, the serum HDL was not significantly different between PCa cases (51 mg/dL) and non-cases (50 mg/dL) in the population.

Statistical analysis included coefficient of determination (R^2^) to test a good-ness-of-fit for linear regression models and Pearson correlation coefficients (r_s_) to examine the strength and direction of the association between the eight EPs and PBs metabolites and numerical variables, as described in the PCa cases (Table 5). Age showed a strong relationship in the linear regression model and a very high positive correlation with BPA (R^2^ of 0.9; r_s_ of 0.9), Triclosan (R^2^ of 0.9; r_s_ of 0.9), Methylparaben, (R^2^ of 0.8; r_s_ of 0.8), Benzophenone-3 (R^2^ of 0.7; r_s_ of 0.8), and Propylparaben (R^2^ of 0.6; r_s_ of 0.8). Bodyweight (BW) recorded fitting for the linear regression model and a high positive correlation with BPA (R^2^ of 0.6; r_s_ of 0.8) and Ethylparaben (R^2^ of 0.5; r_s_ of 0.7). Total cholesterol was determined higher fitting for the linear regression model and a very high positive correlation with BPA (R^2^ of 0.9; r_s_ of 0.9) and Triclosan (R^2^ of 0.7; r_s_ of 0.8). Triglyceride was determined higher fitting for the linear regression model and had a very high positive correlation with BPA (R^2^ of 0.8; r_s_ of 0.9) and Triclosan (R^2^ of 0.8; r_s_ of 0.9), Benzophenone-3 (R^2^ of 0.7; r_s_ of 0.8), Propylparaben (R^2^ of 0.7; r_s_ of 0.8), and both Methylparaben and Ethylparaben (R^2^ of 0.6; r_s_ of 0.8). LDL noted fitting for the linear regression model and a very high positive correlation with BPA (R^2^ of 0.8; r_s_ of 0.9) and Triclosan (R^2^ of 0.8; r_s_ of 0.9), Benzophenone-3 (R^2^ of 0.7; r_s_ of 0.9), and both Methylparaben and Ethylparaben (R^2^ of 0.7; r_s_ of 0.9), and Propylparaben (R^2^ of 0.6; r_s_ of 0.8). HDL showed no fitting in the linear regression model and a negligible correlation with all the metabolites. Butylparaben and 4-tert-octyl phenol did not fit the linear regression model and had an insignificant correlation with all the numeric variables (Table 5).

This study selected the coefficient progression with Schwartz’s Bayesian criterion (SBC) and SBC (or sometimes called Bayesian information criterion, BIC) criterion for model selection. The BIC value increases as model complexity increases, and BIC decreases with the likelihood increase. Therefore, a lower BIC is better. Seventeen variables were examined by applying the stepwise regression procedure (forward and backward selection combination). The final selected model is the equivalent to the forward and backward methods until all the independent variables left in the model are significant or every significant independent variable is added.

**Figure 2:**
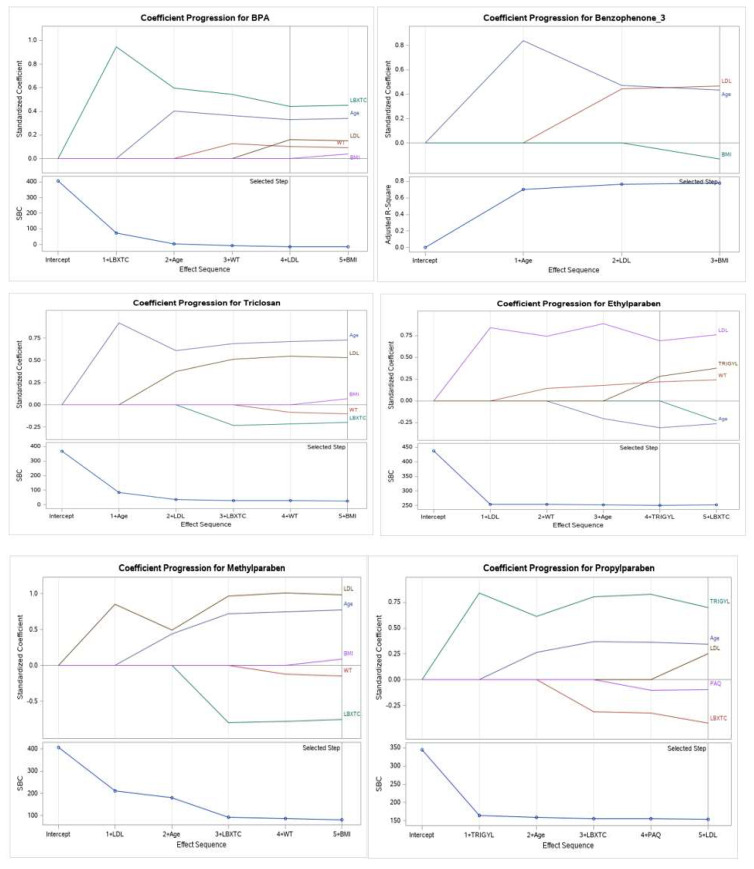
Coefficient progression with Schwartz’s Bayesian criterion (SBC) for PCa cases for the six EPs and parabens metabolites (BPA, Benzophenone−3, Triclosan, Propylparaben, Ethylparaben, and Methylparaben) and PCa for men ≥20 years of age (NHANES 2005–2015), and quantitative and descriptive variables (total cholesterol LDL, triglycerides, age, BMI, weight, and physical activity).

Figure 2 demonstrates that the selected quantitative and descriptive variables for the models using Schwartz’s Bayesian criterion (SBC) showed significant association with some EPs metabolites and PBs with PCa cases. BPA (along with higher values of total cholesterol, age, LDL, weight, and BMI), Triclosan (along with higher age, LDL, BMI, weight, and total cholesterol), Benzophenone-3 (along with more elevated LDL, age, and BMI) associated significantly with PCa outcome. The selected variables for PCa cases connected with environmental PBs metabolites are Ethylparaben (along with higher values of LDL, triglyceride, weight, and age), Methylparaben (along with higher values of LDL, age, BMI, weight, and total cholesterol), and Propylparaben (along with higher values of triglyceride, age, LDL, physical activity, and total cholesterol). No association of variables was observed in the case of 4-tert-octyl phenol and PCa.

For PCa cases, BPA, Benzophenone-3, and Triclosan showed more fitting for a linear regression model and a higher positive correlation with age, total cholesterol, LDL, triglyceride, and weight. For PCa cases, Ethylparaben, Methylparaben, and Propylparaben conferred more fitting for a linear regression model and a higher positive correlation with LDL, triglyceride, total cholesterol, weight, and age. Further, 4-tert-octyl phenol and Butylparaben did not fit the linear regression model and negatively correlated with all the numerical variables.

The three EPs (BPA, Triclosan, and Benzophenone-3) showed higher fit in linear regression models, higher linear relationship, and higher Pearson correlation coefficients of strength and direction of the association with all the numerical variables (except with weight and HDL) in the PCa cases than non-cases. The three PBs (Ethylparaben, Methylparaben, and Propylparaben) showed higher fit in linear regression models, higher liner relationship, and higher Pearson correlation coefficients strength and direction of the association with all the numerical variables (except with weight and HDL) in the PCa cases than non-cases. The six above metabolites showed higher fit in linear regression models, a higher linear relationship, and higher Pearson correlation coefficients of strength and direction of the association with a weight in PCa cases than in non-cases.

### 2.4. Odds Ratio and Confidence Intervals

The estimated odds ratios (OR) and 95% confidence intervals (OR, 95% CI) for the risk of having PCa, and the eight EPs and PBs metabolites are presented in Table 6. After adjusting for age, weight, total cholesterol, HDL, triglyceride, and LDL, BPA, Triclosan, Benzophenone-3, Propylparaben, Ethylparaben, and Methylparaben were observed to be significantly associated with PCa. The strongest associations with PCa risk among the metabolites were found for Propylparaben (OR of 1.7, 95% CI: 1.56–1.76), and BPA (OR of 1.5, 95% CI: 1.43–1.56).

### 2.5. Gene Enrichment Analysis, PPI Network, Hub Gene Identification, and Validation Analysis

**Figure 3:**
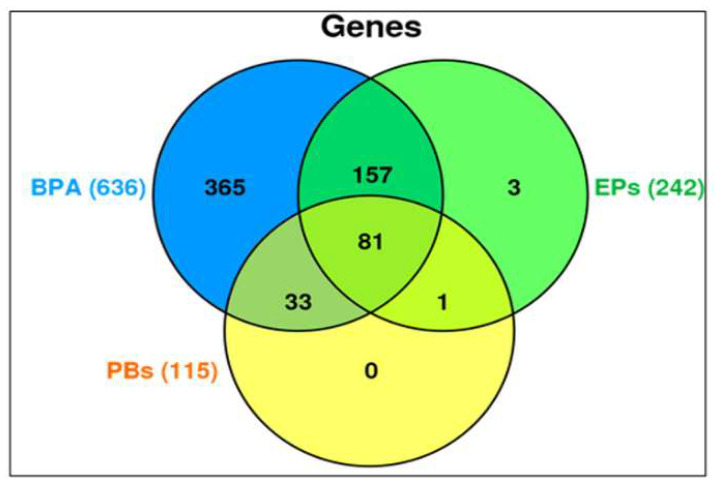
Venn diagram generated from CTD analyses of genes that are influenced by the exposure of BPA, EPs, and PBs separately or in combinations associated with the PCa outcomes. BPA was separated from EP chemical groups because, separately, BPA influenced the highest number (636 genes).

Our CTD analysis showed that there were 993 differentially influenced (upregulated or downregulated) genes aligned with PCa outcome. The Venn diagram in Figure 3 shows the genes influenced separately by BPA (636 genes), EPs (242 genes), and PBs (115 genes). The overlapping influenced genes between (BPA and EP), and (BPA and PB), and (EP and PB) are (157 genes), and (33 genes), and (one gene), respectively. BPA was separated from EP chemical groups because BPA influenced the highest number of genes separately (636 genes). Nonetheless, together, the EPs and PBs investigated in this study influenced 81 common genes that were associated with PCa.

The GO enrichment and KEGG pathway analysis were completed on the overlapping genes utilizing the DAVID database, including the Gene Association Diseases Database (GAD), BP, CC, MF, transcription factor binding sites (TFBS), and KEGG pathway. The statistically significant (*p* < 0.05) top ten terms of each enrichment analysis are assembled in Tables S7 and S8. In Table S8, the gene ontology and pathway enrichment analysis were significantly enriched in BP. They included their response to estradiol, positive regulation of transcription from RNA polymerase II promoter, and their response to the drug. These 81 overlapping genes were significantly enriched to suggest their presence in the various CCs of the protein complexes, nucleoplasm, and extracellular space. Moreover, these 81 genes were evaluated for their MF by enriching their significant transcription factor binding, sequence-specific DNA binding, and steroid-binding. The KEGG signaling pathway analysis showed that the genes were markedly enriched for the pathways in cancer, PCa, Hepatitis B, colorectal cancer, and HIF-1 signaling. The transcription factor binding sites enrichment analysis of these 81 genes in PCa by UCSC-TFBS showed the top five TFs with the number of their target genes, as shown in Table 7.

1. TATA Box: *p*-Value = 7.29 × 10^−4^, connected with 51 genes
2. CEBPB: *p*-Value = 7.29 × 10^−4^, connected with 51 genes
3. E2F: *p*-Value = 2.80 × 10^−3^, connected with 52 genes.
4. NFKAPPAB: *p*-Value = 3.06 × 10^−3^, connected with 36 genes
5. SRY: *p*-Value 3.46 × 10^−3^, combined with 40 genes.

The GAD enrichment analysis of 81 overlapping common gene functions showed that 35 genes (CDKN1A, CDKN1B, LHB, SERPINE1, KLK3, KLK2, NR3C1, CYP19A1, CYP17A1, CASP9, CCND1, PLAU, CDH1, MYC, DNMT3B, CYP1B1, CD14, NCOA2, NOS2, EGF, UGT2B15, IGF1, ESR1, ESR2, GNMT, VEGFA, AR, IL1B, SELENOP, CYP1A1, BCL2, ID3, PPARA, SHBG, TP53) have the highest significance value (1.54 × 10^−27^) in their association with PCa.

**Figure 4:**
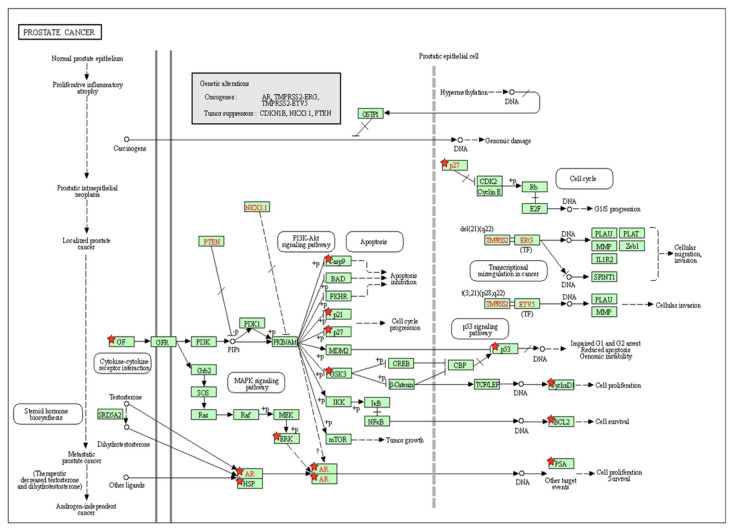
The identification of key molecular alterations in PCa signaling KEGG pathway. Eight out of 13 genes (BCL2, CASP9, CCND1, CDKN1A, EGF, HSP90B1, MAPK3, and TP53) were identified and marked with the red star by KEGG signaling pathway for PCa.

**Figure 5:**
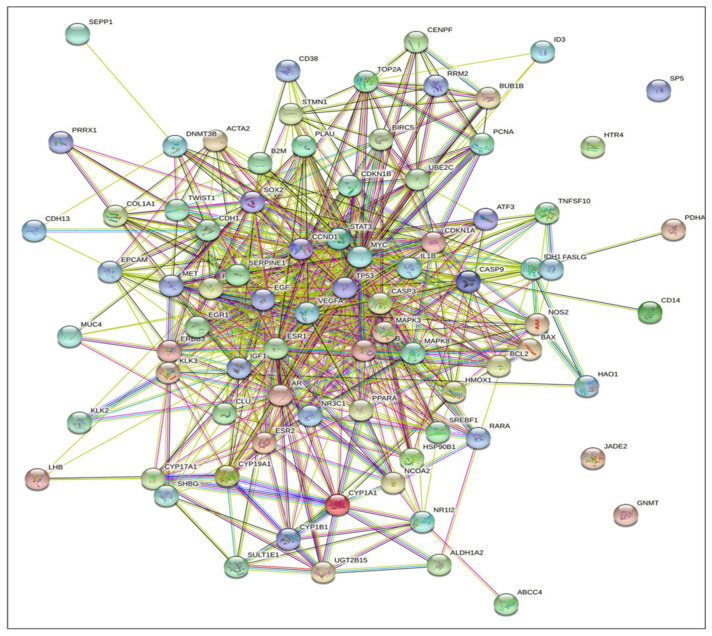
STRING database analysis of the protein–protein interactions (PPI) networks for the functional enrichment analysis yielded EPs and PBs influenced 81 overlapping genes (nodes) and the number of 698 edges with degree > 7, and PPI enrichment *p*-value < 1.0 × 10^−16^.

The KEGG signaling pathway enrichment analysis for PCa for EP and PB influenced 81 overlapping genes and also showed that there are 25 genes (GSK3B, CDKN1A, CDKN1B, KLK3, FASLG, HSP90B1, CASP9, MAPK8, CCND1, CDH1, MYC, CASP3, MAPK3, NOS2, EGF, STAT3, IGF1, VEGFA, AR, BCL2, RARA, BAX, BIRC5, MET, TP53) that were markedly enriched with pathways in cancer (Table S8). In addition, there are 13 genes (BCL2, AR, CASP9, CCND1, CDKN1A, CDKN1B, EGF, GSK3B, HSP90B1, IGF1, KLK3, MAPK3, and TP53) that were markedly enriched for PCa pathways (Table S8). Moreover, in the KEGG analysis, eight out of the 13 genes (BCL2, CASP9, CCND1, CDKN1A, EGF, HSP90B1, MAPK3, and TP53) were identified and marked with the red star by the KEGG signaling pathway for PCa (Figure 4). The analysis of the KEGG pathway of PCa aligned key genes involved in carcinogen defenses (GSTP1), growth-factor-signaling pathways (NKX3.1, PTEN, and p27), and androgen receptor (AR), which are critical determinants of the PCa phenotype [46]. A protein–protein interactions (PPI) network was created utilizing the STRING database tool. There were 81 nodes and 698 edges in the network, with a local clustering coefficient of 0.37, average node degree 4.53, and PPI enrichment *p*-value < 1.0 × 10^−16^, which demonstrated the PPI enrichment for the network was statistically significant (Figure 5). Then, we used Cytoscape to identify hub genes among the 81 influenced EP and PB overlapping genes.

**Figure 6:**
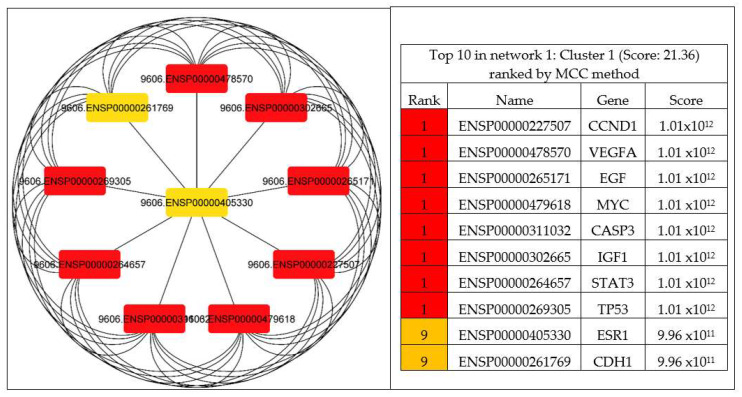
Identified hub genes by CytoHubba among the 81 overlapping genes. The top eleven nodes are shown with a color scheme from red (highly important) to orange (important), and the top eleven genes in the PPI network were calculated by MCC.

The CytoHubba was utilized to explore important genes in the PPI network by implementing DNMC and MCC to identify the hub genes of PCa. The results discovered nine genes ranked as one (CCND1, VEGFA, EGF, MYC, CASP3, IGF1, STAT3, and TP53) and two genes ranked as nine (ESR1 and CDH1) were considered as the most significant (1.01 × 10^12^) hub genes in the network, as shown in Figure 6.

We also compared our findings with another 11 studies, which have also used gene datasets to identify the hub genes associated with the PCa. The following list summarizes that only two genes (CDH1 and VEGFA) aligned with the previously published studies.

1. IKZF1, PPM1A, FBP1, SMCHD1, ALPL, CASP5, PYHIN1, DAPK1, and CASP8 [52].
2. KLK3, CDH1, KLK2, FOXA1, and EPCAM [48]
3. RPS21, FOXO1, BIRC5, POLR2H, RPL22L1, and NPM1 [49]
4. EPCAM, TWIST1, CD38, and VEGFA [46].
5. LMNB1, TK1, RACGAP1, and ZWINT [53].
6. IGF2, GATA5, F10, CFI, AGTR1, FOXA1, BZRAP1-AS1, and KRT8 [54].
7. PIK3R1, BIRC5, ITGB4, RRM2, TOP2A, ANXA1, LPAR1, and ITGB8 [55].
8. CDH1, BMP2, NKX3-1, PPARG, and PRKAR2B [56].
9. CDCA8, CDCA5, UBE2C, and TK1 [57].
10. PPFIA2, PTPRT, PTPRR, PRR16, CHRM2, KRT23, CYP3A4, CYP3A7, and DPYS, and DUOXA1 [58].
11. TSPAN1, BMPR1B, FOXA1, STEAP1, RCAN3, S100P, LYZ, SCGB3A1, IL8, and DPT [47].

**Figure 7:**
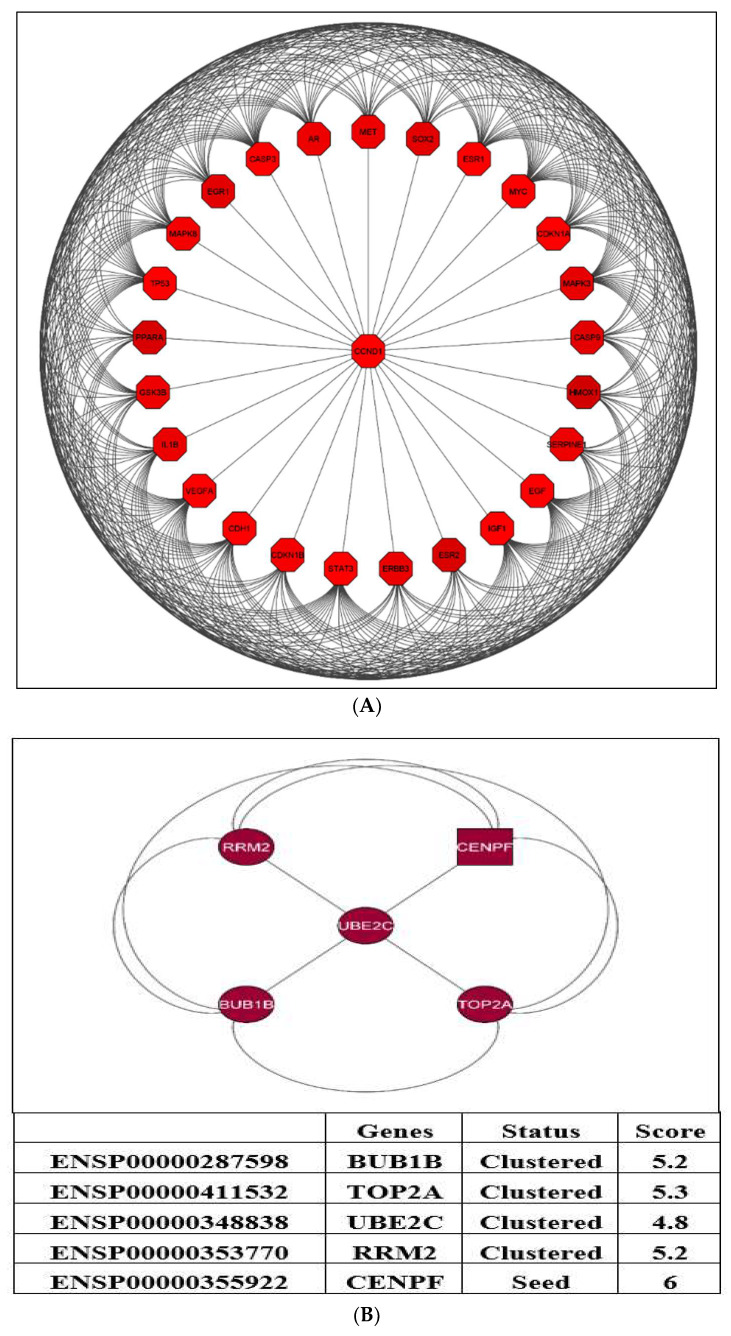
The two modules were generated from the PPI network. (**A**) Module-1: associated with a score of 21.36 and includes 25 genes (nodes) and 175 edges. (**B**) Module-2: associated with a score of 5; consists of five genes (nodes) and 10 edges.

The results from the MCODE analysis of 81 genes show two modules. With a score of 21.36, Module-1 included 25 genes (nodes) and 175 edges, and Module-2, with a score of 5, included five genes (nodes) and 11 edges. A PPI network was then created by using five genes (nodes) and ten edges, which is displayed in Figure 7. The genes identified in the Module-2 network (top five clusters) containing five clustered proteins (BUB1B, TOP2A, UBE2C, RRM2, and CENPF) were represented as hub genes for the subsequent analysis. The MCODE research for Module-2 of the top five clusters containing seed protein is highlighted in the square node shape responsible for forming the clusters.

**Figure 8:**
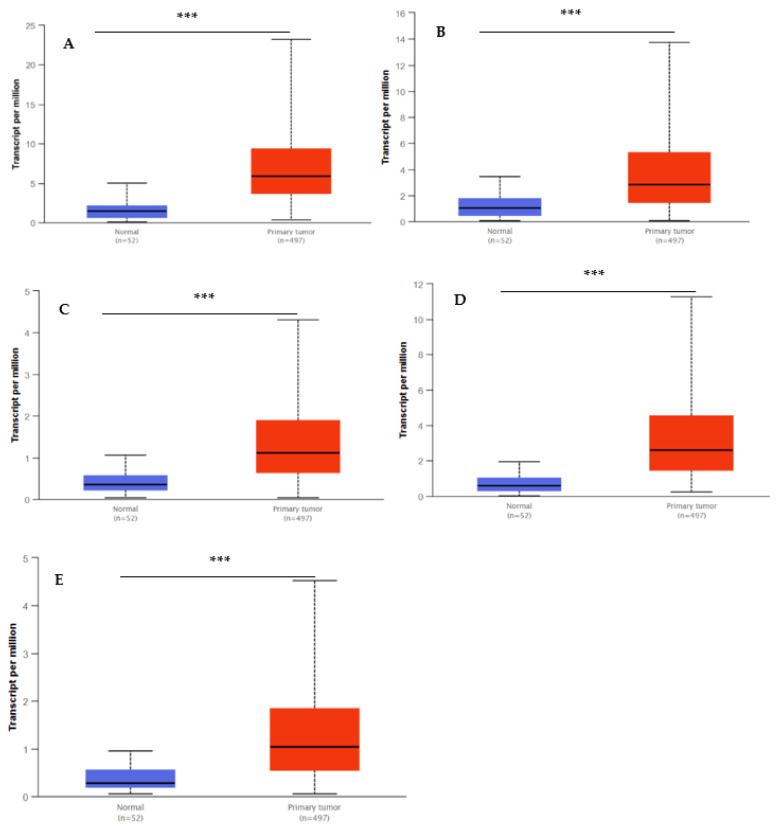
Box whisker plots indicating the expression of five hub genes in PCa samples. (**A**) UBE2C; (**B**), TOP2A; (**C**) BUB1B; (**D**) RRM2; and (**E**) CENPF were identified by MCODE and verified at the protein level by UALCAN, which came from the TCGA project. Data are mean ± SE. *** *p* < 0.001.

**Figure 9:**
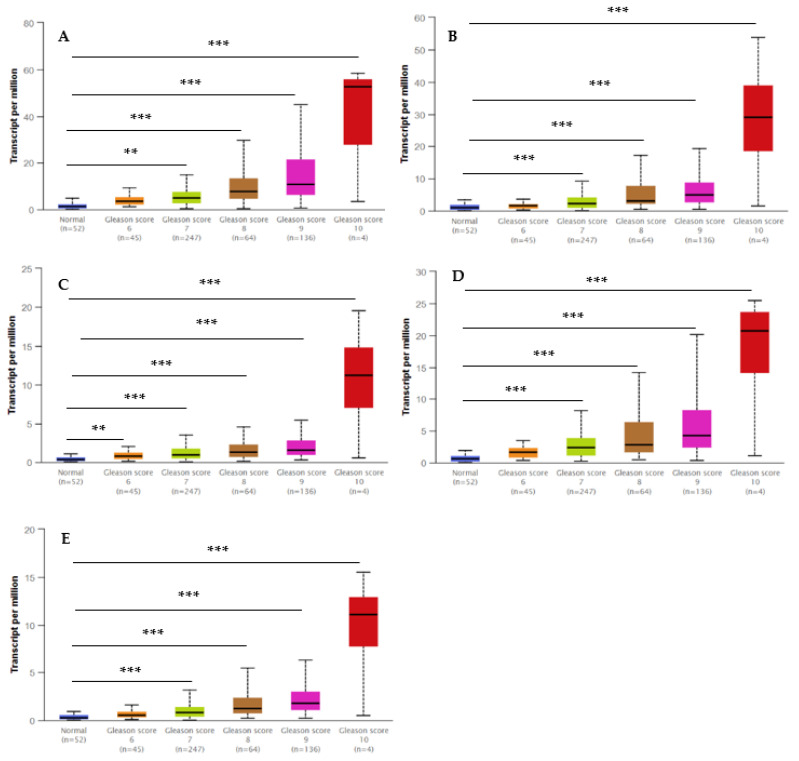
Box whisker plots indicating the different Gleason scores (6, 7, 8, 9, and 10) in the PCa and the normal tissues from the TCGA dataset. Significantly high levels of five hub genes in PCa samples are shown in panels: (**A**) UBE2C; (**B**), TOP2A; (**C**) BUB1B; (**D**) RRM2; and (**E**) CENPF. Data are mean ± SE. ** *p* < 0.05; *** *p* < 0.001.

The UALCAN database was utilized to validate the transcript expression levels of five hub genes in 549 samples derived from the TCGA project for PCa (the threshold was set as |log2FC| = 1, *p* < 0.05). The statistical samples contained 497 PCa and 52 normal samples. As indicated in Figure 8, there was an obvious statistical significance (*p* < 0.001) for all the hub genes’ expression levels (BUB1B, TOP2A, UBE2C, RRM2, and CENPF) in PCa compared to normal samples. Moreover, Figure 9 shows the positive correlations between candidate hub genes and Gleason’s score of the PCa samples. High expression levels of the five hub genes were associated with advanced stages (Gleason score ≥ 8) and recurrence, and the hub genes were significantly higher in the most aggressive PCa tissues (Gleason score = 10). All the hub genes were highly expressed, with the statistical significance (*p* < 0.001) for Gleason score = 9 with the highest aggressiveness and the poorest prognosis.

## 3. Discussion

Numerous studies have suggested that EPs and PBs may have mixed or single effects (agonists or antagonists) on sex hormones [59]. In animal models, exposures to specific EPs and PBs are associated with the pathogenic effects in obesity [22], thyroid dysfunction [60], breast cancer [61], and PCa [62]. It is suggested that the primary route of EP and PB exposure is by ingestion via the food supply chain. For example, canned food is recognized as the primary source of BPA exposure [29,63]. Despite the challenges associated with the half-life and the measurements of the conjugated or free forms of EP [5,42,43,63] and PB [5,19,20,43], urine is by far the most accepted biological sample to evaluate their exposure. Previous studies have also reported EP [5,18,25] and PB [5,64] concentration in the urine samples as indicators of daily exposures in male populations. The current research assessed the levels of four EP and four PB metabolites in the urine samples of the male population accessed from the NHANES data (2005 to 2015). This cross-sectional study of US men >20 years demonstrated higher urine levels of EP (BPA, Benzophenone-3, 4-Tert-Octyl Phenol, and Triclosan) and PB (Propylparaben, Ethylparaben, and Methylparaben) were significantly associated with an increased risk of PCa in the US men’s population. In our cross-sectional study, the three EPs and four PBs also showed an increased association with the categorical variables of age and individual body weights in the PCa cases. The fact that our study shows the association of PCa cases with the higher levels of EP and PB found in the urine samples of US men during the ten years (2005–2015—five cycles of NHANES data) is an important finding, especially when it shows a significantly higher association in the higher age group of the PCa cases. It is a consistent observation that, in the higher age group, higher levels of EPs and PBs were found to be associated with the self-reported PCa cases in all five cycles of NHANES data (2005–2015) analyzed in this study. The study also alleviated the selection biases in the observation of the significant association of EPs and PBs with PCa in higher age groups by bootstrapping the data (data not shown here) in different age groups.

In the follow-up analysis, we considered specific co-variates that may have increased the higher risks of EP and PB exposures in the PCa cases. Upon analysis of the NHANES (2005–2015) questionnaires filled out by the subjects in this study, high BPA levels with PCa were significantly associated with higher BMI and those who consumed canned and frozen food frequently. This observation aligns with studies that have also shown that consumption of fast food and canned food places the older population at a higher risk for weight gain and cancers [17,63,65]. Differences between race/ethnicity can be associated with variations in food habits in adults, especially men, utilizing unhealthy dietary behaviors. Their food choices and consumption of unhealthy foods, such as processed foods, frozen foods, canned foods, and soft drinks, are associated with higher BPA exposures [17,65]. Benzophenone-3 was significantly higher in the PCa cases when compared with non-cases. Furthermore, it was noted in this study that Benzophenone-3, a chemical detected in the plastic packaging and personal products (sunscreen and lotion), has the highest metabolite level (21.2 ng/mL) present in the urine samples of the men reported having been diagnosed with PCa. Another study has reported that Benzophenone-3 was detectable in most urine specimens and in >30% of the blood samples of the populations reported to have been diagnosed with cancers [5,42]. We found that Benzophenone-3 is present in significantly higher levels in older age, non-Hispanic Whites than non-Hispanic Blacks, and those who eat canned and frozen food regularly than those who do not eat canned and frozen food. Other research findings suggest that regular personal care products are an important source of exposure to phenols and diethyl phthalate in adults and provide data on children’s exposure to selected phenols and phthalates [66].

High Triclosan concentrations in adipose tissue have been reported in the older subjects, with higher BMI and overweight related to the consumption of unhealthy foods [26,60]. Still, these studies did not analyze any particular disease outcome. The urine level of Triclosan in our study was also significantly higher in the higher age group and higher BMI associated with PCa cases. PB chemicals are extensively used in various food products as antimicrobial preservatives and pharmaceuticals [5,20,24,43]. Therefore, we again assumed ingestion as the primary route of exposure. PB metabolites were detectable more commonly in the urine samples than in the serum and human milk [5]. An earlier study [5] showed that Propylparaben and Methylparaben had the highest urine levels, with mean values of 9.1 and 43.9 mg/dL, respectively. Moreover, Ethyl, Methyl, and Butylparaben were inversely correlated with BMI ≥30 kg/m^2^ (obesity) and positively associated with age. These results corroborate earlier studies [5,19,24]. Using NHANES data (2005–2015), our investigation found that all the urine levels of evaluated PB, except Ethylparaben, were significantly higher in the PCa cases than the non-cases. Besides, PB chemicals were higher in the non-Hispanic White men than non-Hispanic Black men. Similar results were obtained in the non-Hispanic White men and non-Hispanic Black men on the urinary PB levels from exposure studies with NHANES (2005–2006 and 2013–2014) data cycles [19,67]. These studies did not conduct a disease association analysis. PB chemicals, however, act as EDCs, and the ability of parabens to enable multiple cancer hallmarks in human breast epithelials have been documented [68].

Observation of the associated higher levels of EP and PB in the PCa cases with higher BMI and older age led us to investigate the total lipid profiles of the study population in the NHANES data (2005–2015). In a meta-analysis of cross-sectional data from NHANES (2003–2014), no associations were found between urinary BPA and the five different lipids (low-density lipoprotein (LDL), high-density lipoprotein (HDL), total cholesterol (TC), triglycerides (TG), and apolipoprotein B (ApoB)) variables when investigated in both children (≤17 years old) and adults (≥18 years old) [16]. This study also did not conduct any disease association analysis. However, in our study, except for HDL, we found that TC, LDL, and TG were significantly higher (*p* < 0.0001) in PCa cases than non-cases in the subjects with higher BMI and older age. Earlier studies have shown that LDL, TC, and TG were positively associated with PCa, breast cancer, and colon cancer [69]. Furthermore, other prospective research presented a positive association between the three lipid profiles and PCa incidence [70]. Moreover, some prospective studies have proposed that high LDL and total cholesterol may increase the risk of PCa [69], and cholesterol-lowering medications have been associated with a decreased risk of advanced PCa in observational research [71]. Therefore, our inference that higher lipid levels in the US men population increase the PCa cases corroborates the disease association studies. On the other hand, Propylparaben concentration with a 40% and 95% CI: 3, 90, and Ethylparaben with 63% and 95% CI: 2, 86 were linked to the higher prevalence of metabolic syndrome among men and women, respectively [72]. A study that did not include EP exposures presents similar results observed in this study that total cholesterol concentration, LDL, and triglycerides were positively linked with PCa in men [69]. The study was conducted on 1,189,719 Korean adults (24,475 women and 53,944 men were diagnosed with cancers), which concluded that high total cholesterol (>240 mg/dL) was strongly linked with PCa (HR, 1.24; 95% CI, 1.07 to 1.44). Possibly, a study mainly designed for longitudinal research combined with EPs and PBs (free and conjugated) measurements will suggest any possible role of their (EPs and PBs) existing body burden on the PCa outcome.

It is important to understand the influence of the combinations of the descriptive and quantitative covariates along with the EPs and PBs association with PCa outcome. As shown in Figure 2, we examined 17 variables by using the stepwise regression procedure (forward and backward selection combination) for the coefficient progression (SBC or BIC) criterion for model selection with EP and PB. The selection model number One showed that total cholesterol, age, LDL, weight, and BMI were selected for three EPs (BPA, Triclosan, and Benzophenone-3) associated with PCa cases. The selection model number Two showed that total LDL, triglyceride, weight, age, and BMI were chosen for the last model of three PBs (Propylparaben, Ethylparaben, and Methylparaben) for PCa cases (Figure 2). PB metabolites are also known as alkyl esters of para-hydroxybenzoic acid, and they interact with lipids, especially triglycerides in adipose tissue, and influence lipid metabolism. Earlier studies suggest that urinary excretion of propylparaben and methylparaben are associated with a lower concentration of triglycerides [19]. An experiment conducted in the model systems showed that the formation of animal lipids might explain the impact of PB cytotoxic activity in the cell membranes (phosphatidylcholine). It also depends on the type of lipid, solution concentration, and chemical composition [73]. Therefore, the influence of PB on the model systems comprised of human lipids may explain these cytotoxic activities. One research noted a significant association between adipose tissue measures and PB biomarker concentrations among the US general population NHANES data between 2007 and 2014. The ORs (95% CI) for obesity increase in methylparaben concentrations were 0.64 (95% CI: 0.55, 0.73) for adults, and relationships were more noticeable among men [20]. In our research, the highest concentration of BPA, Propylparaben, and Ethylparaben appeared to be the most notable risk in men with PCa in adjusted logistic regression models for age, weight, total cholesterol, HDL, triglyceride, and LDL and adjusted logistic regression models for eight metabolites. However, 4-tert-octyl phenol and Propylparaben exhibited the most notable risk in men with PCa in the unadjusted logistic regression model (Table 6). The urinary BPA level is considered a prognostic biomarker in PCa samples, and low-dose BPA may contribute to the prostate’s neoplastic transmutation. A study involving 60 PCa patients found higher levels of urinary BPA (creatinine-adjusted) in PCa patients (5.74 mg/g [95% CI; 2.63, 12.51]) than in controls (1.43 mg/g [95% CI; 0.70, 2.88]) (*p* = 0.012) [12].

The association of EP and PB with PCa then raises the question of how they (EP and PB) may be contributing to the etiology of PCa. Conventionally, in vitro studies or animal models can be employed to investigate the effects of different doses of EP and PB, alone or in their combinations, to map the genetic pathways on the development and prognosis of PCa. This method may allow extrapolating the EP and PB risk assessment results to apply in humans, still with a degree of uncertainty. On the contrary, we used in silico curated works of thousands of researchers who have investigated the effects of EP and PB at the molecular level in mammalian cell cultures or mammalian models to compare and describe the potential role of EP and PB in the PCa etiology, especially when our results show more relevance to the older population. In addition, we have utilized numerous available public databases and web-based bioinformatics that have separately mapped the networks and pathways of carcinogenesis of different tissues, including PCa. It is important because, despite the progress achieved in understanding the molecular processes and the progression in PCa, it remains a high risk for morbidity and mortality in the male population of the US [7]. With the new findings, it is becoming increasingly clear that PCa is a complex disease, and many endogenous and exogenous risk factors may play a role in the progression of PCa. EP and PB may be risk factors that may have additive effects on the etiology of PCa in US men. Therefore, in the next step, the study included identifying responsive genes to EP and PB exposures employing the CTD. We understand that a human subject is simultaneously exposed to many chemicals in real time. While retaining all the chemical exposure at a time is beyond the scope of this work, we have integrated BPA, EP, and PB, which are the focus of our study to identify genes that are responsive to their exposures. As presented earlier, our integrative approach determined genes with the possibility of their interactions with EP and PB that may be essential to the development of PCa. We identified 636, 242, and 115 genes associated with BPA, EPs, PBs exposure, and connected with PCa, respectively, which were discovered using mammalian systems. It was interesting that unique genes were influenced (overexpressed or under-expressed) by BPA, EP, and PB separately. Still, 81 genes were commonly affected (Figure 3) and mapped in the PCa prognosis on the CTD. Further, the available in silico curated public databases have allowed us to investigate a network of genes to obtain a somewhat comprehensive view of PCa etiology in response to EP and PB exposures in mammalian systems.

In the next step, our results illustrated that EP and PB gene ontology and pathway enrichment analysis influenced 81 overlapping genes aligned with their curated function in PCa for core genes that were increased in different categories of biological processes (BP). They included a response to estradiol and positive regulation of transcription from RNA polymerase II promoter, suggesting their role in the PCa progression via different BP. The androgen receptor is the typical target for PCa detection and therapy, and estrogens and their receptors have also been implicated in the development of PCa [46]. The RNA polymerase II promoter is important for the transcription of DNA [49] and was identified in various studies for being involved in BP enrichment analysis in different cancers, such as breast cancer [74], ovarian cancer [75], and liver cancer [76]. Our results showed, for the KEGG signaling pathway analysis: the genes markedly enriched cancer, PCa, Hepatitis B, colorectal cancer, and the HIF-1 signaling pathway. Activated HIF-1 plays a critical function in the adaptive reactions of the tumor cells to differences in oxygen via transcriptional activation of 100 downstream genes, which control the essential BP needed for tumor survival and progression [77]. The analysis of the GO terms, pathway enrichment, and KEGG pathway demonstrate these 81 (different numbers for different BP; please see Table S8) EP- and PB-influenced genes to be involved in the BP, which appear to be directly or indirectly engaged in the development and progression of PCa.

When this identified set of EPs and PBs that influenced 81 overlapping genes was subjected to transcription factor binding sites enrichment analyses, specifically for the PCa pathway, the most significant top five TFs were as follows:

1. TATA-box-binding protein (TBA)—which is a general TF that operates at the core of the DNA-binding multiprotein factor and regulates the transcriptional activity of RNA polymerase II [78].
2. CEBPB—an important TF that regulates the expression of genes involved in immune and inflammatory responses [79].
3. E2F is a TF that binds with DNA for genes involved in the cell cycle and DNA replication, which can mediate cell proliferation and TP53 mediated apoptosis [80].
4. SRY is a TF that regulates the genetic switch to male development and is affected by various gene regulations, including promoter activation or repression [81].
5. NFKAPPAB (NF-kappa-B) is a TF that is found in almost all cell types and is affected in multiple biological processes, such as immunity, cell growth, inflammation, differentiation, apoptosis, and tumorigenesis [82].

However, the question remains as to how 81 EP- and PB-influenced genes may be working together in a PCa network. We used Cytoscape, a web-based bioinformatics software, to visualize molecular interaction networks. The CytoHubba plugin screened for core genes following a PCa prognosis analysis that was comparable to previous studies [46,48,74,83]. Through the CytoHubba and PPI network, CCND1, VEGFA, EGF, MYC, CASP3, IGF1, STAT3, TP53, ESR1, and CDH1 were identified as hub genes and considered to be playing an important role(s) in PCa progression. Interestingly, when the hub genes identified by the 11 eligible studies published were compared with those specified in the current study, only two genes (CDH1 and VEGFA) were found to be common. The result then indicates that the other eight genes, which are EP- and PB-responsive (CCND1, EGF, MYC, CASP3, IGF1, STAT3, TP53, ESR1), are possibly unique to our studies contributing to the PCa prognosis.

MCODE specified with Module-1 was associated with 26 genes, and Module-2 was associated with five genes and 11 edges. The analysis of the selected five genes from Module-2 (BUB1B, TOP2A, UBE2C, RRM2, and CENPF) was further validated by using PCa samples and normal tissue from TCGA. A significant increase in the gene expression levels of all five hub genes in PCa tumors compared to normal tissues suggests their role in the PCa. BUB1B is a critical mitotic checkpoint kinase. BUB1B is identified as the top-scoring kinase by RNA interaction. It is possible that a novel antimitotic target involves impaired spindle checkpoint function in any form of cancer, including PCa [51,57]. DNA topoisomerase II alpha (TOP2A) and ribonucleotide reductase regulatory subunit M2 (RRM2) were discovered to be the most significant in PPI network analysis and survival analysis in liver cancer and for PCa [76]. TOP2A is a critical nuclear enzyme for chromosome condensation, DNA mitosis, and cell division. Moreover, TOP2A may be utilized as a biomarker that indicates poor prognosis and may function as a treatment target for cervical cancer [83]. TOP2A has been suggested to directly interact with P53, a well-known tumor suppressor protein. In Ref. [84], it was reported that UBE2C, TOP2A, and CCNB1 were associated with PCa prognosis and higher expression in PCa samples than in normal tissues. RRM2 is associated with poor overall survival of breast cancer patients and can become a useful target for diagnosing and treating patients [74]. Ubiquitin-conjugating enzyme E2C (UBE2C) was highly expressed in PCa compared with normal tissue in TCGA; the expression was also more highly associated with the Gleason score (>7) of PCa, which plays as an independent prognostic factor of PCa, and recreated a critical role in the pathway of PCa (WNT-β-catenin signaling pathway and NOTCH signaling pathway) [84]. Moreover, UBE2C was reported high in breast cancer, indicated to be an independent prognostic factor connected with the recurrence and death, and associated with a shorter survival period of breast cancer patients. Therefore, it is regarded as a possible therapeutic target for breast cancer [74]. Centromere protein F (CENPF) gene encodes a protein that associates with the nuclear matrix component during the G2 phase, including cell growth and protein synthesis during mitosis. CENPF is a potentially applicable candidate for diagnosing and treating cervical cancer [83]. CENPF was one of 41 hub genes closely connected with Gleason scores and the “T” stage in PCa [85]. CENPF is involved with 20 candidate genes to be risk factors related to aggressive PCa and overexpressed consistently in PCa samples’ carcinogenesis at the TCGA [84]. The overall identified genes by different gene analysis tools for 81 overlapping genes associated with PCa, EPs, and PBs were summarized in Table S7. The biological functions of the five hub genes closely associated with the progression of PCa were summarized in Table S9.

The observation of different Gleason scores (6, 7, 8, 9, and 10) and normal tissues in the TCGA samples by expression of the hub genes depending on the Gleason score method is illustrated in Figure 9. A low Gleason score (≤6) shows a low prognosis without the risk of metastasis, whereas a high Gleason score (>8) is associated with an increased risk of metastasis. These hub genes may be responsive to other exposures or cellular pathways leading to cancer. Nonetheless, our study indicates that EP and PB exposures may be working via these hub genes to contribute directly or indirectly to the progression of PCa.

Our study has limitations as it is a cross-sectional study with self-reported PCa data on NHANES (2005–2015) without the information on the family history of hormonal cancers. Only the men who reported no cancer diagnosis (non-cases group) or PCa diagnosis (cases group) were covered in our research population. It is also highly challenging to establish the food chain exposure as the primary route of human exposures of the selected EPs and PBs. Nonetheless, using the available in silico information and integrated bioinformatic tools, we identified a set of EP- and PB-responsive hub genes of the PCa pathway that can be further investigated in the biological models for their use as diagnostic or therapeutic targets.

## 4. Materials and Methods

### 4.1. Study Design and Population

The NHANES is continuous cross-sectional population-based datasets. It is nationally representative by collecting data and sampling from the noninstitutionalized U.S population, confidential, voluntary participants, and civilian populations. NHANES considers multistage datasets, complex, and probability of sampling and subgroups designs [86,87]. It is a health survey for the standard population, which approximately includes 5000 participants. It is carried out in a two-year cycle. Participants also perform household questionnaires that involve demographic information, diet and nutrition data, medical and health conditions, and collected biological samples (blood and urine), which are obtained through mobile examinations center (MEC) [88]. The medical questionnaire has been conducted yearly since 1999 by CDC/National Center for Health Statistics (NCHS) to evaluate the U.S. population’s general health and nutritional situation. The NCHS institutional review committee reviews and approves all NHANES protocols, and all participants provided written, informed consent, and child permission before any data or sample collection [21,88]. We used sample weighting scheme data from 2005–2006, 2007–2008, 2009–2010, 2011–2012, and 2013–2014 study cycles. Our study samples are limited and included only those males ≥20 years of age who completed demographic information, medical health conditions questionnaires, and provided biological samples (urine samples) and examinations measurements at the MEC. The specified data collection cycles are as follows (years, PCa-cases, and non-ceases): (2005–2006, 25, 725), (2007–2008, 33, 855), (2009- 2010, 31, 939), (2011–2012, 31, 1034) and (2013–2014, 32, 887) and are summarized in Figure S4.

### 4.2. Medical Health Questionnaire

Medical conditions (self-reported cancers) were collected utilizing the medical questionnaires in NHANES. The participants’ ≥20 years old responded to “Have you ever been told by a doctor or other health professionals that you had cancer or malignancy of any kind? Men who responded “yes” were consequently asked, “What kind of cancer was it”? and “What was your age at diagnosis?” According to the questions, the person was required to report physician-diagnosed cancers. There was no additional validation of the cancer diagnosis in these data. Only men who reported no cancer diagnosis (non-cases group) or PCa diagnosis (cases group) were covered in our research population. Other kinds of cancers were eliminated from this analysis. The authors of Refs. [86,88] have also used PCa diagnosis (yes/no) as a categorical dependent variable. Data analyses investigated the association of the concentration of urinary EPs and PBs metabolites (obtained from laboratory data), demographic data, and self-reported health outcomes (gathered from questionnaire data) of male participants ≥20 years of age.

### 4.3. EP and EB Exposure Assessment

We integrated three independent sources, the CPCat (Chemical and Product Categories), the EPA Toxicity Forecaster (ToxCast), and the FDA/Total Diet Study (TDS) through the Center for Food Safety and Applied Nutrition (CFSAN), as shown in Figure S5, to compile a list of chemicals used in the food supply in the United States. Urinary EP and PB were measured on a randomly selected subsample covering one-third of NHANES participants aged six years and older. NHANES uses a sensitive technique for measuring EP and PB that was developed in 2005 [88]. The procedure utilizes online solid-phase extraction (SPE) linked to High-performance liquid chromatography (HPLC) and tandem mass spectrometry (HPLC–MS/MS). Applying isotopically labeled internal measures, the detection limits in 100 μL of urine are 0.1–2.3 (ng/mL), sufficient for estimating urinary levels of EPs and PBs in exposed subjects [88]. Urine specimens are prepared, stored, and shipped to the Division of Laboratory Sciences, National Center for Environmental Health/CDC for analysis. Vials are saved frozen at −20 °C until they are sent to the National Center for Environmental Health for measurement [88]. The QA/AC protocols in NHANES satisfy the 1988 Clinical Laboratory Improvement Act. The NCHS and contract consultants conduct QC measurements through unscheduled visits. Laboratory personnel work with qualified persons for equipment calibration and specimen samples preparation. The employees go through the annual training to assure that needed skill levels are maintained. We analyzed recorded levels of EPs and PBs in the urine samples and self-reported PCa cases in the participants ≥20 years of age during five NHANES survey cycles (2005–2015). The EPs and PBs compounds and their metabolites that were analyzed in the urine samples of our study are (1) Bisphenol A (URXBPH), (2) 2-Hydroxy-4-metoxybenzophenone (Benzophenone-3, URXBP3), (3) 4-Tert-octylphenol (URX4TO), (4) 2,4,4′-Trichloro-2′-hydroxyphenyl ether (Triclosan, URXTRS), (5) Butylparaben (URXBUP), (6) Ethylparaben (URXEPB), (7) Methylparaben (URXMPB), and (8) Propylparaben (URXPPB).

### 4.4. Sample Measurements and Limits of Detection (LOD)

A detection limit variable is presented for all EPs and PBs in the datasets. For chemicals measured in urine, LOD computations were conducted utilizing the chemical concentration shown per urine volume. LOD values may change by survey year because of improvements in analytical techniques, while most LODs were constant for all the analytes in the dataset. Two variables are given for each of these analytes. The variables were identified by (URDXXXLC), which shows whether the result was under the LOD, and (URXXXX), which presents the analytic result for that analyte, which was above LOD. Therefore, there are two values given (0) to indicate that the result was >LOD and (1) to suggest that the result was <LOD. For metabolites with analytic effects less than the LOD, a claimed fill value was assigned in the analyte results area and called a lower limit of detection (LLOD) and (LLOD/√ (2 & 2)).

### 4.5. Statistical Analysis

Our analyses used male participants ≥20 years of age only with available data for EP and PB levels, demographics, and medical health data for all five NHANES survey cycles. All urinary compounds and metabolites for EP and PB were log-transformed and creatinine corrected to satisfy normality assumptions. According to the NCHS guidelines, all measures were weighted to provide accurate national estimates [88]. Chi-square and *t*-test statistics (depending on the variables) were conducted to compare the means and distribution of covariates by PCa status. Logistic regression models were applied to estimate the association and investigate the risk between PCa occurrence, urinary levels of EPs and PBs, and the selected covariables by calculating ORs and their 95% CIs. All statistical analyses were performed using SAS Studio for Windows (SAS version 9.4 software), and *p*-values < 0.05 were recognized statistically significant (we used a 5% significance level).

### 4.6. Covariates

The potential covariates were obtained from the questionnaire interviews (self-reported) or collected as laboratory measurements. The possible variables were inputted as numerical variables and as categorical variables. At the assessment time, age is the only variable entered as numerical (years) and categorical (three groups of age). The study also alleviated the selection biases in the observation of the significant association of EPs and PBs with PCa in higher age groups by bootstrapping the data (data not shown here) for different age groups. Body mass index (BMI) was calculated for participants as weight (kg)/height meters squared (m^2^) and then rounded one decimal place (dp). Participants reported race/ethnicity, family income, education level, birth in the US, physical activity, eating frozen food in the past 30 days, alcohol use, smoking status, and liver diseases, including cancer and kidney disease. The categorical variables are described with questions provided to participants and divided into groups in Table S10. Serum samples were collected from participants at the mobile examinations center (MEC) to analyze blood lipid levels. On the MEC, total cholesterol (BXTC), low-density lipoprotein (LDL), high-density lipoprotein (HDL), and triglycerides (TRIGLY) were analyzed by enzymatic assay. LDL levels on MEC were calculated from determined amounts of BXTC, TRIGLY, and HDL depending on the equation ([LDL] = [BXTC] − [HDL] − [TRIGLY/5]), which is efficient for individuals with BXTC concentration < 400 mg/dL (Table S11). The research strategy’s flowchart for the selection process of outcome, years, chemicals, and variables from the NHANES dataset was summarized in Figure S6.

### 4.7. Genes Associated with PCa Progression

The next step included identifying genes that are influenced (underexpression or overexpression) by EP and PB exposures using the Comparative Toxicogenomic Database (CTD). Biocurators at CTD manually curate gene–disease connections, chemical–disease relationships, and chemical–gene interactions from the literature [89]. Our search criteria included: Disease: PCa, Chemicals (EPs, BPA, 4-tert-Octyl phenol, 2,4,4′-Trichloro-2′-hydroxyphenyl ether (Triclosan), 2-Hydroxy-4-metoxybenzophenone (Benzophenone-3), PBs, methylparaben, Ethylparaben, Propylparaben, and Butylparaben). For the gene query, we looked for the genes associated with PCa, EPs, and PBs. For Chemical–Gene Interaction Query, we searched for EPs and PBs associated with PCa. Data showing curated association with the PCa, EPs, and PBs were downloaded, filtered, sorted with only human samples, and were cross-referenced on PubMed database. We conducted a functional and pathway enrichment analysis by using Database for Annotation, Visualization, and Integrated Discovery (DAVID.6.8). DAVID (https://david.ncifcrf.gov/, accessed on 18 December 2021) is a functional enrichment tool that provides biological descriptions of gene datasets and proteomic research from high-throughput sequencing [90].

We also used Kyoto Encyclopedia of Genes and Genomes (KEGG), which is a database used for high-level functions of the biological system, molecular-level data generated by genome sequencing, and other high-throughput experimental technologies (https://www.genome.jp/kegg/, accessed on 31 December 2021) [91]. The gene ontology (GO) was used to align biological process (BP), molecular function (MF), and cellular component (CC) analysis (http://www.geneontology.org, accessed on 31 December 2021) [92] of the identified genes with the criterion for significance for *p*-value of < 0.05. Together, the GO and KEGG pathway analysis was used to associate genes with their potential biological functions and their roles in PCa pathways.

We implemented the Search Tool for the Retrieval of Interacting Genes (STRING) to build direct and indirect protein–protein interaction networks analysis. STRING is an online database tool (http://string-db.org/, accessed on 31 December 2021) that functions as an access point for the more reasonable interpretation of relationships between various proteins on a genome-wide scale. It is helpful to understand the protein functions [93]. We combined our analysis criteria under the following conditions: average node degree 17.2, local clustering coefficient 0.644, PPI enrichment *p*-value < 1.0 × 10^−16^, the minimum required interaction score of 0.4, active interaction, human species, experiments, and gene fusion databases, and co-recurrence.

The Molecular Complex Detection (MCODE) plug-in in Cytoscape (version 3.9.0) software was utilized to examine the significant modules and determine potential functional modules in the protein–protein interaction (PPI) network with the following parameters (MCODE scores > 7, degree cutoff = 2, node score cutoff = 0.1, Max depth = 100 and k-score = 2) [94]. The hub genes were then estimated by various methods with CytoHubba plug-in in Cytoscape (version 3.9.0) software. The CytoHubba is widely used to study the most important node (genes) in various biological networks. It is used to investigate the most significant node in various biological networks. CytoHubba contained eleven topological analysis procedures, while, in this study, we utilized ((MCC: Maximal Clique Centrality) and (DNMC: Degree, Density of Maximum Neighborhood Component)) to determinate candidate hub gene of PCa [95]. We collected our results on Radiality, Closeness, Degree, EcCentricity, Edge, Percolated Component, Bottleneck, Maximum Neighborhood Component (MNC), Maximal Clique Centrality (MCC), Clustering Coefficient (CC), Stress and Betweenness (SB), Density of Maximum Neighborhood Component (DMNC).

The relative expression of hub genes between PCa and normal samples and Gleason grade and recurrence situation was examined using the online UALCAN database. The analysis of relative expression of the hub genes in PCa was conducted utilizing UALCAN (http://ualcan.path.uab.edu, accessed on 19 December 2021) [96]. UALCAN is an interactive web resource that is user-friendly and comprehensive for investigating cancer OMICS data. UALCAN is developed to access cancer OMICS data (TCGA, MET500, CPTAC, and CBTTC), and it allows the users to identify biomarkers or conduct in silico validation of potential genes of interest. Once we identified the hub genes, we validated their differential expressions by using TCGA samples of PCa tumors and normal samples.

## 5. Conclusions

To the best of our knowledge, this is the first research that has comprehensively used NHANES human data collected between 2005 and 2015 (five cycles of data collection) to associate EPs and PBs, assumed to be mostly present in the food supply chain, as risk factors for PCa in the US men population. We selected these chemicals monitored by the EPA and FDA in the food products and NHANES (CDC) in the human urine samples for over a decade. They are also on the priority list of the US national toxicology program [21]. We demonstrate a significant association of higher levels of EPs and PBs monitored in the urine samples of the US men with reported prostate cancer cases. We also report that higher levels of EP and PB in the urine samples are consistent with the increase in the severity, especially in the older population of 65 years and older, with higher BMI and high lipid concentrations in the blood. The association of EP and PB and PCa effect was correlated with some selected variables, such as age, weight, BMI, and lipids concentration, as supported by previous studies [43,97,98,99,100,101], enhancing the PCa risks in the subjects. In this risk assessment approach, we innovatively used the curated in silico information on differential gene expressions, pathways, and genetic networks of PCa on various databases: TCGA, GO, GEO, KEGG, DAVID, CTD, Cytoscape, and UALCAN. We validated the hub genes’ expression from 81 identified genes using TCGA data from PCa cases to strengthen the observations on overlapping DEG in response to EP and PB by CTD analysis. In this study, these identified hub genes, BUB1B, TOP2A, UBE2C, RRM2, and CENPF, shed light on the underlying molecular mechanisms triggered by EP and PB exposures, which may have additive effects on the PCa etiology. This approach offers an alternative to using animals and cellular studies to generate hypotheses and narrow down the focus to elevate this vital area of research to the next level. Environmental health risk assessment prevents and mitigates public exposures to hazards [102]. We believe that this research presents a strong case demonstrating the association of EP and PB, a group of endocrine-disrupting chemicals that may have additive effects on prostate cancer prognosis in US men. The association of EP and PB exposures with PCa shown in this study and the gene networks generated indicates that prostate carcinogenesis is not a linear pathology, and the hub genes potentially accommodate exposures of risk factors, including metabolic, co-morbidity, age, or lifestyle, to exacerbate the severity of the disease. Concomitantly, we suggest that the severity of the disease may be affected by the triggering of these hub genes during the prostate cancer etiology. The study demonstrates the EP and PB exposure, alone or in combination, as risk factors for PCa in US men (sampled in the five cycles of NHANES survey data), and it has shown an innovative approach to identify hub genes in the PCa etiology. These findings open intervention channels to reduce and replace the use of EPs and PBs in the food supply to mitigate their exposure(s) and help develop robust molecular diagnostics or therapeutic targets for PCa.

## Figures and Tables

**Table 1:** Descriptive statistics (categorical variables) for PCa status and selected covariates among men ≥ 20 years of age; NHANES 2005–2015.

Variables	Male Population (*n* = 4592)	
	PCa Cases	Non-Cases
**Total Population (*n*, %)**	(152, 3.3%)	(4440, 96.7%)
**Age (years, %)**		
20–49	9 (0.2%)	2195 (47.8%)
50–69	61 (1.4%)	1481 (32.3%)
≥70	81 (1.7%)	764 (16.6%)
**BMI (kg/m^2^, %)**		
≤25	35 (0.8%)	1763 (38.4%)
25 to 30	42 (0.9%)	1362 (29.7%)
≥30	75 (1.6%)	1315 (28.6%)
**Race/ethnicity (*n*, %)**		
Non-Hispanic White	80 (1.7%)	2115 (46.1%)
Non-Hispanic Black	46 (1.0%)	1387 (30.2%)
Others	26 (0.6%)	938 (20.4%)
**Income (Annual Family Income) (*n*, %)**		
≤$24,999	47 (1.0%)	1243 (27.1%)
$25,000 to $ $54,999	56 (1.2%)	1423 (31.0%)
$55,000 to $74,999	22 (0.5%)	882 (19.2%)
≥$74,999	27 (0.6%)	892 (19.4%)
**Education (*n*, %)**		
≤12th grade	78 (1.7%)	2444 (53.2%)
>12th grade	74 (1.6%)	1999 (43.5%)
**Alcohol use (*n*, %)**		
Yes	115 (2.5%)	3050 (66.4%)
No	37 (0.8%)	1390 (30.3%)
**Ever smoked (*n*, %)**		
Yes	99 (2.2%)	2218 (48.3%)
No	53 (1.1%)	2222 (48.4%)
**US Birth (*n*, %)**		
Yes	124 (2.7%)	3307 (72.0%)
No	28 (0.6%)	1133 (24.7%)
**Physical activity (*n*, %)**		
Yes	19 (0.4%)	2025 (44.1%)
No	133 (2.9%)	2415 (52.6%)
**Eat frozen food (*n*, %)**		
Yes	131 (2.9%)	1624 (35.4%)
No	21 (0.5%)	2816 (61.3%)
**Liver diseases (*n*, %)**		
Yes	0 (0.0%)	2 (0.04%)
No	152 (3.3%)	4438 (96.7%)
**Kidney disease (*n*, %)**		
Yes	1 (0.02%)	3 (0.1%)
No	151 (3.28)	4437 (96.6%)

**Table 2:** Descriptive statistics (numerical variables) PCa status and selected covariates among men ≥ 20 years of age; NHANES 2005–2015.

Variables	Male Population (*n* = 4592)	
	PCa	Non-Cases
Total Population (*n*, %)	(152, 3.0%)	(4440, 97.0%)
Age (years; mean ± se)	69.0 ± 0.9	50.0 ± 0.3
Bodyweight (kg; mean ± se)	86.0 ± 1.3	76.0 ± 0.3
Serum Total Cholesterol (mg/dL, mean ± se)	228.0 ± 6.0	179.0 ± 0.6
Serum HDL (mg/dL, mean ± se)	51.0 ± 1.2	50.0 ± 0.2
Serum LDL (mg/dL, mean ± se)	199.0 ± 7.0	106.0 ± 0.5
Serum Triglycerides (mg/dL, mean ± se)	246.0 ± 8.3	98.0 ± 0.7

**Table 3:** EPs and PBs levels (ng/mL) in the urine samples of men ≥ 20 years of age with concentrations ≥ LOD; NHANES 2005–2015.

BPA							
	Number	Mean	Std Dev	Std Err	Minimum	Maximum	*p*-value
Non-cases	4440	2.5	2.4	0.04	0.28	16.3	<0.05
Cases	152	8.0	3.8	0.3	1.1	17.6	
Benzophenone-3							
	Number	Mean	Std Dev	Std Err	Minimum	Maximum	*p*-value
Non-cases	4440	7.1	6.5	0.1	0.28	26.1	<0.05
Cases	152	21.2	3.7	0.3	12.0	29.0	
4-Tert-Octyl Phenol							
	Number	Mean	Std Dev	Std Err	Minimum	Maximum	*p*-value
Non-cases	4440	0.16	0.13	0.001	0.14	3.8	<0.05
Cases	152	0.34	0.5	0.04	0.14	3.5	
Triclosan							
	Number	Mean	Std Dev	Std Err	Minimum	Maximum	*p*-value
Non-cases	4440	5.9	4.5	0.01	1.6	20.8	<0.05
Cases	152	17.0	3.3	0.3	10.0	23.6	
Butylparaben							
	Number	Mean	Std Dev	Std Err	Minimum	Maximum	*p*-value
Non-cases	4440	0.71	1.6	0.03	0.14	19.9	0.2645
Cases	152	0.60	0.9	0.07	0.14	3.8	
Ethylparaben							
	Number	Mean	Std Dev	Std Err	Minimum	Maximum	*p*-value
Non-cases	4440	2.3	3.0	0.05	0.71	18.2	<0.05
Cases	152	12.7	4.2	0.3	6.00	19.5	
Methylparaben							
	Number	Mean	Std Dev	Std Err	Minimum	Maximum	*p*-value
Non-cases	4440	9.4	4.3	0.07	0.71	22.6	<0.05
Cases	152	16.6	3.8	0.3	8.10	25.0	
Propylparaben							
	Number	Mean	Std Dev	Std Err	Minimum	Maximum	*p*-value
Non-cases	4440	3.6	3.9	0.05	0.14	17.8	<0.05
Cases	152	15.7	3.0	0.03	6.9	21.3	

**Table 4:** Age, weight, and lipid profile levels (mg/dL) in the male population; NHANES 2005–2015.

Variables	Male Population (*n* = 4592)		
	PCa Cases	Non-Cases	*p*-Values
Total Population (*n*, %)	(152, 3.7%)	(4440, 96.3%)	
Age (years; mean ± se)	69.0 ± 0.9	50.0 ± 0.27	<0.05
Bodyweight (kg; mean ± se)	86.0 ± 1.3	76.0 ± 0.3	<0.05
Serum Total Cholesterol (mg/dL, mean ± se)	228 ± 0.9	179 ± 0.6	<0.05
Serum HDL (mg/dL, mean ± se)	51 ± 1.0	50 ± 0.2	0.1928
Serum LDL (mg/dL, mean ± se)	199 ± 1.0	106 ± 0.5	<0.05
Serum Triglycerides (mg/dL, mean ± se)	247 ± 0.8	98 ± 0.7	<0.05

**Table 5:** Coefficient of determination (R^2^) and Pearson correlation coefficients (r_s_) between environmental phenols and parabens and numeric variables for PCa cases population; NHANES 2005–2015.

	Age		Weight		Total Cholesterol		HDL		TRYGLY		LDL	
	R^2^	r_s_	R^2^	r_s_	R^2^	r_s_	R^2^	r_s_	R^2^	r_s_	R^2^	r_s_
Analyte												
BPA	0.9	0.9	0.6	0.8	0.9	0.9	0.1	−0.2	0.8	0.9	0.8	0.9
4-tert-octyl phenol	0.003	0.01	0.01	0.01	0.02	0.2	0.006	−0.01	0.001	0.03	0.001	−0.03
Triclosan	0.9	0.9	0.4	0.6	0.7	0.8	0.03	−0.1	0.8	0.9	0.8	0.9
Benzophenone-3	0.7	0.8	0.4	0.6	0.6	0.8	0.001	0.01	0.7	0.8	0.7	0.9
Propyl paraben	0.6	0.8	0.3	0.6	0.5	0.7	0.003	−0.06	0.7	0.8	0.6	0.8
Butyl paraben	0.06	−0.3	0.001	−0.07	0.05	−0.2	0.001	0.03	0.03	−0.2	0.04	−0.2
Ethyl paraben	0.4	0.7	0.5	0.7	0.6	0.7	0.001	−0.07	0.6	0.8	0.7	0.9
Methyl paraben	0.8	0.8	0.4	0.6	0.5	0.7	0.01	−0.1	0.6	0.8	0.7	0.9

**Table 6:** Estimated odds ratios (95% CI) for risk of having PCa cases by the concentration of EP and PB levels in the urine samples of men ≥ 20 years of age; NHANES 2005–2015.

Analytes	PCa Cases (*n* = 152)		
	Adjusted OR (95% CI) ^1^	Adjusted OR (95% CI) ^2^	Unadjusted OR (95% CI)
BPA	1.4 (1.33–1.53) ^a^	1.3 (1.13–1.58) ^a^	1.5 (1.43–1.56) ^a^
4-tert-octyl phenol	0.9 (2.95–11.59)	0.8 (0.63- 1.99)	0.7 (0.53–1.57)
Triclosan	1.3 (1.26–1.40) ^a^	1.4 (1.22–1.50) ^a^	1.5 (1.41–1.55) ^a^
Benzophenone-3	1.3 (1.21–1.33) ^a^	1.2 (1.11–1.31) ^a^	1.4 (1.36–1.50) ^a^
Propyl paraben	1.5 (1.39–1.60) ^a^	1.6 (1.38–1.71) ^a^	1.7 (1.56–1.76) ^a^
Butyl paraben	1.0 (0.87–1.21)	0.5 (0.33–0.73) ^a^	0.97 (0.86–1.08)
Ethyl paraben	1.4 (1.34–1.49) ^a^	1.3 (1.14–1.38) ^a^	1.5 (1.44–1.56) ^a^
Methyl paraben	1.3 (1.18–1.34) ^a^	0.95 (0.84–1.08)	1.5 (1.44–1.60) ^a^

^1^ Adjusted for age, weight, total cholesterol, HDL, TRYGLY, and LDL. ^2^ Adjusted for eight metabolites. ^a^ *p* < 0.05.

**Table 7:** Transcription factor binding sites enrichment analysis of the EP and PB influenced 81 overlapping genes in PCa (UCSC-TFBS: University of California Santa Cruz (UCSC) Genome Browser—Transcription factor binding sites). The highly significant-top five TF with the number of their target genes is shown in this table.

	TF	Count	*p*-Value	Genes
1	TATA	52	7.29 × 10^−4^	FOXA1, TOP2A, GSK3B, CDKN1B, SERPINE1, BUB1B, FASLG, NR3C1, SOX2, C CND1, PLAU, MYC, CASP3, DNMT3B, B2M, ABCC4, SREBF1, UGT2B15, AR, ALDH1A2, IL1B, SELENOP, RARA, CDH13, PPARA, MET, TP53, ATF3, PCNA, LHB, NR1I2, TWIST1, CYP19A1, MAPK8, ERBB3, SULT1E1, HAO1, EGR1, PDHA1, PRRX1, EGF, STAT3, IGF1, ESR1, ESR2, COL1A1, CENPF, CYP1A1, BCL2, SP5, ID3, SHBG
2	CEBPB	51	7.29 × 10^−4^	FOXA1, GSK3B, CDKN1B, SERPINE1, BUB1B, FASLG, JADE2, HTR4, NR3C1, CLU, SOX2, CDH1, MYC, DNMT3B, NCOA2, ABCC4, SREBF1, AR, ALDH1A2, IL1B, SELENOP, RARA, CDH13, PPARA, MET, ATF3, LHB, NR1I2, KLK3, KLK2, HSP90B1, CYP17A1, ERBB3, HMOX1, HAO1, CD14, MAPK3, EGR1, PRRX1, NOS2, EGF, UBE2C, IDH1, STAT3, IGF1, ESR1, ESR2, CENPF, CYP1A1, BCL2, SHBG
3	E2F	52	2.80 × 10^−3^	FOXA1, TOP2A, GSK3B, CDKN1A, CDKN1B, SERPINE1, BUB1B, JADE2, NR3C1, CLU, SOX2, CCND1, MYC, STMN1, DNMT3B, CYP1B1, NCOA2, ABCC4, SREBF1, AR, ALDH1A2, RARA, CDH13, TP53, ATF3, PCNA, TWIST1, CYP19A1, HSP90B1, CYP17A1, ERBB3, SULT1E1, HMOX1, HAO1, CD14, MUC4, MAPK3, EGR1, RRM2, PRRX1, EGF, UBE2C, STAT3, IGF1, ESR1, ESR2, GNMT, CYP1A1, BCL2, BAX, SP5, ID3
4	NFKAPPAB	36	3.06 × 10^−3^	TOP2A, CDKN1A, CDKN1B, TWIST1, FASLG, JADE2, NR3C1, HTR4, CYP19A1, HSP90B1, CASP9, ERBB3, CCND1, PLAU, CDH1, MYC, TNFSF10, DNMT3B, HMOX1, B2M, MAPK3, SREBF1, NCOA2, EGR1, NOS2, EGF, STAT3, ESR1, AR, ALDH1A2, IL1B, BCL2, RARA, SP5, CDH13, TP53
5	SRY	40	3.46 × 10^−4^	FOXA1, TOP2A, GSK3B, CDKN1B, NR1I2, SERPINE1, TWIST1, BUB1B, JADE2, NR3C1, HTR4, CYP19A1, HSP90B1, CYP17A1, SOX2, MYC, CYP1B1, MUC4, MAPK3, SREBF1, NCOA2, ABCC4, EGR1, PRRX1, IDH1, STAT3, IGF1, ESR1, ESR2, VEGFA, AR, CENPF, ALDH1A2, IL1B, SELENOP, BCL2, RARA, CDH13, PPARA, MET

## Data Availability

The datasets generated during the current study are available from the corresponding author on reasonable request.

## Supplementary Materials

The following supporting information can be downloaded at: https://www.mdpi.com/article/10.3390/ijms23073679/s1.

## Abbreviations

BIC	Bayesian information criterion
BMI	Body mass index
BP	Biological process
BPA	Bisphenol A
BUB1B	BUB1 mitotic checkpoint serine/threonine kinase B
CDC	Disease Control and Prevention measures
CC	Cellular component
CENPF	Centromere protein F
CTD	Comparative Toxicology Database
DAVID	Annotation, Visualization, and Integrated Discovery
DEGs	Differentially expressed genes
DNMC	Degree, Density of Maximum Neighborhood Component
EDCs	Endocrine-disrupting chemicals
EP	Environmental phenols
EPA	Environmental Protection Agency
FDA	Food and Drug Administration
GAD	Gene Association Diseases Database
GO	Gene ontology
HDL	High-density lipoprotein
KEGG	Kyoto Encyclopedia of Genes and Genomes
LOD	Limits of detection
LDL	Low-density lipoprotein
MCC	Maximal clique centrality
MCODE	Molecular Complex Detection
MEC	Mobile examinations center
MF	Molecular function
NCHS	National Center for Health Statistics
NHANES	National Health and Nutrition Examination Survey
OR	Odds ratios
PBs	Parabens
PCa	Prostate cancer
POPs	Persistent organic pollutants
PPI	Protein–protein interaction
PSA	Prostate-specific antigen
RRM2	Ribonucleotide reductase regulatory subunit M2
SBC	Schwartz’s Bayesian criterion
STRING	Search Tool for the Retrieval of Interacting Genes/Proteins
TBA	TATA-box-binding protein
TF	Transcription factor
TSCA	Toxic Substances Control Act
TOP2A	Topoisomerase II alpha
UBE2C	Ubiquitin-conjugating enzyme E2 C
