# Supplementary material for: Environmental Phenol and Paraben Exposure Risks and Their Potential Influence on the Gene Expression Involved in the Prognosis of Prostate Cancer
Source: Int J Mol Sci. 2022 Mar 27;23(7):3679. doi: 10.3390/ijms23073679 (PMC8998918; doi:10.3390/ijms23073679)
Supplement: Supplementary file 1 [file ijms-23-03679-s001.zip › Supplementary Materials-S1 (figures and tables).pdf]

Supplementary materials

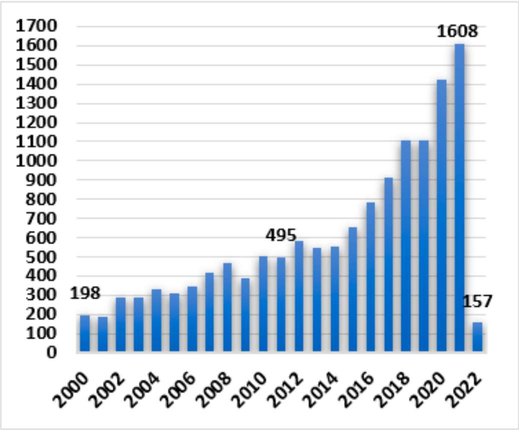

Figure S1: Articles on PubMed as of January 2022 when searched for Toxicity AND Food AND (Environmental AND Phenol) OR Paraben OR (Endocrine AND Disrupt). we found 1608 articles in 2021.

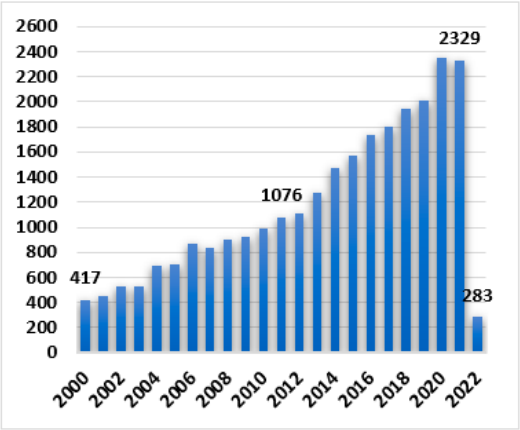

Figure S2: Articles on PubMed as of January 2022 when searched for Toxicity AND food AND Review. We searched on PubMed with specific keywords [(Review, Toxicity, Food)] and [(Toxicity AND Food AND (Environmental AND Phenol) OR Paraben OR (Endocrine AND Disrupt)], we found 2329 articles in 2021.

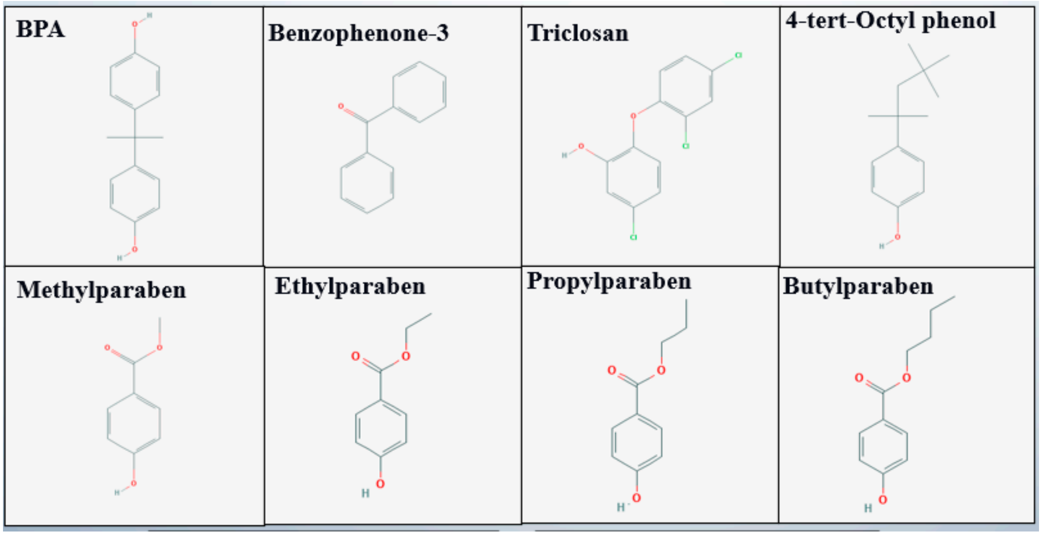

Figure S3: The chemical structure of the EPs and PBs chemicals.

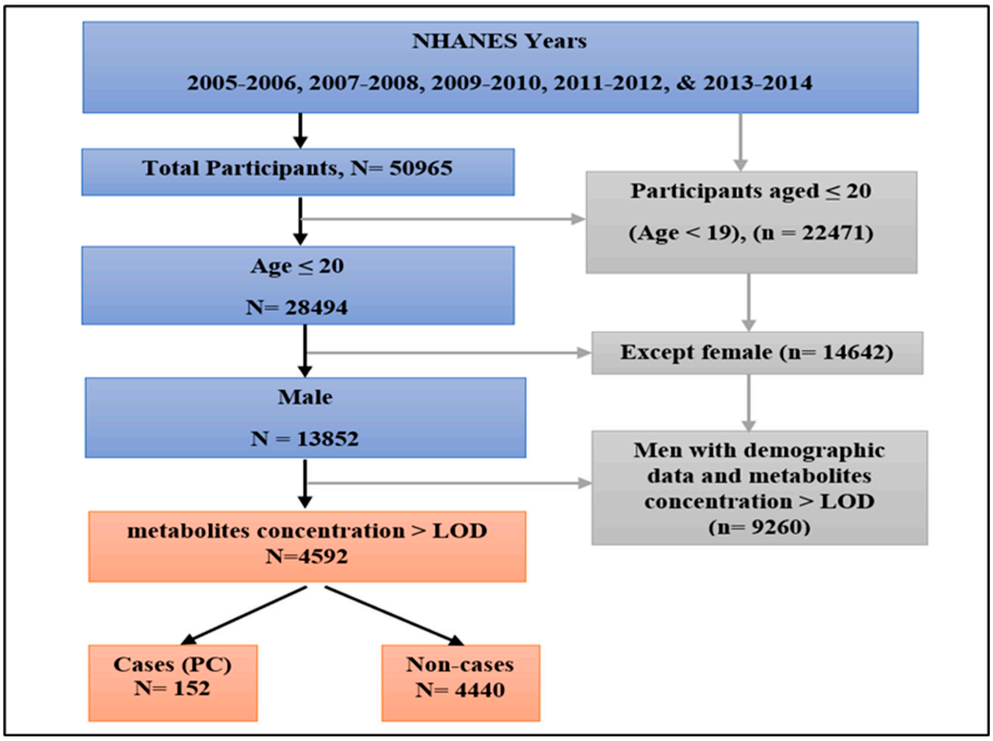

Figure S4: The flowchart of participants selection from NHANES (2005-2006, 2007-2008, 2009- 2010, 2011-2012, and 2013-2014).

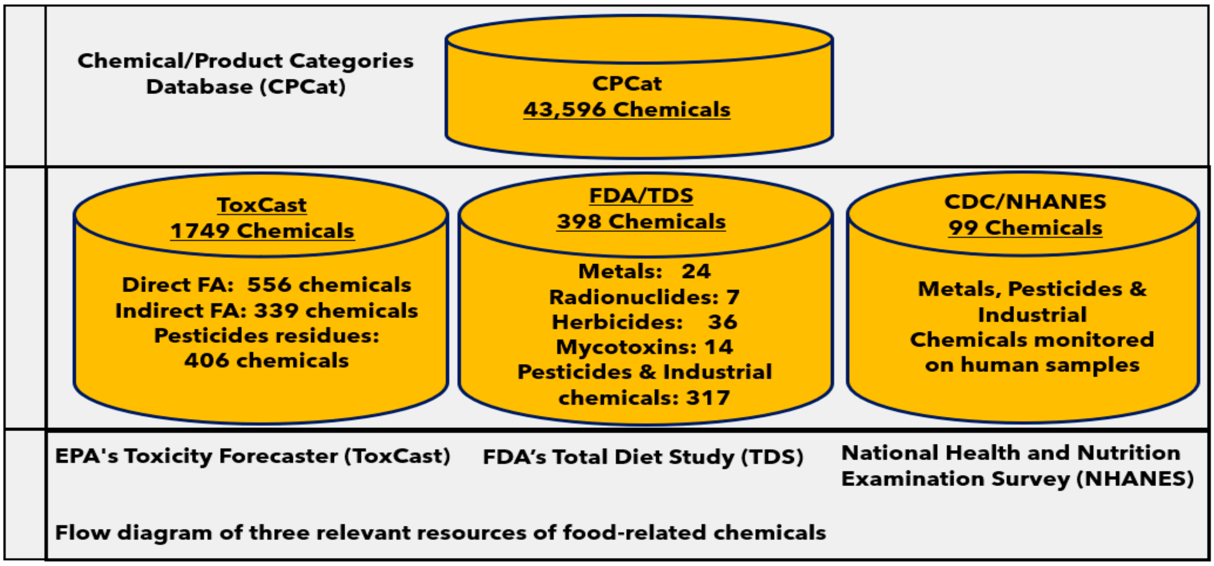

Figure S5: Electronic research of the following databases: The CPCat (Chemical and Product Categories), the EPA Toxicity Forecaster (ToxCast), and The FDA /Total Diet Study (TDS) through the Center for Food Safety and Applied Nutrition (CFSAN). *CPCat is a database including information for more than 43,500 chemicals categorized by*

their usage and function. CPCat was constructed by combining various and different data sources, including ACToR, UseDB, DfE, Dow, DrugBank, 2006 IUR, KemI, and NICNAS. EPA Toxicity Forecaster (ToxCast) creates a database and predictive patterns on thousands of compounds. ToxCast applies high-throughput screening techniques and computational toxicology programs to sort, list, and prioritize chemicals. ToxCast database has nearly 2000 chemicals from various sources, such as food additives, industrial chemicals, and consumer products. In the ToxCast, compounds were categorized and classified into three food classes: (a) pesticide residues, (b) indirect food additives, (c) direct food additives. The FDA has carried the Total Diet Study (TDS) through the Center for Food Safety and Applied Nutrition (CFSAN). The TDS and CFSAN are designed to monitor and observe the US food supply for levels of toxic chemical contaminants such as toxic chemicals, pesticide residues, and industrial elements. The CFSAN study conducted from 2003 to 2014 targeted different compounds, analytes, and their numbers includes metals, radionuclides, mycotoxins, herbicides, pesticides, and industrial chemicals 24, 7, 14, 36, and 317, respectively.

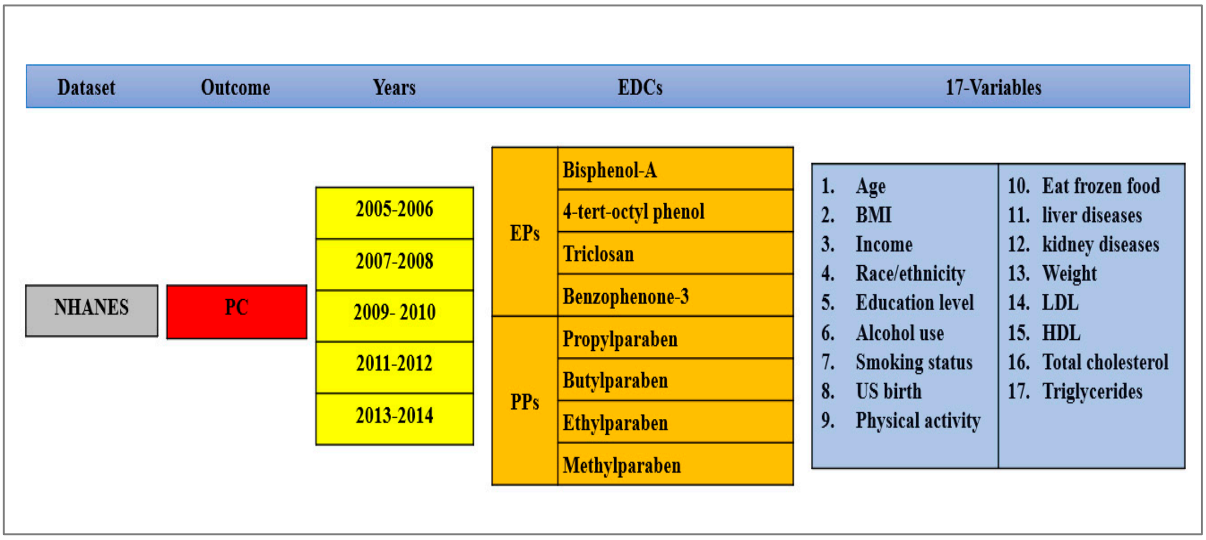

Figure S6: The research strategy's flowchart for the selection process of the outcome, years, chemicals, and variables from the NHANES dataset.

Tables

Table S1: Analysis of significant levels of BPA (ng/ml) associated with the PCa cases and non-cases; NHANES 2005-2015 (* P < 0.05).

Variables	Non-Cases (N=4440)		PCa cases (N=152)		
	**n**	**Mean ± se**	**n**	**Mean ± se**	
Age					**P-values**
20 – 49	2195	2.5±0.3	9*	2.6±0.3	0.9942
50 – 69	1481	2.5±0.1	61*	5.0±0.2	<.0001
≥ 70	764	2.4±0.3	81*	10.7±0.3	<.0001
BMI					
≤ 25	1763	2.3±0.06	35*	8.1±0.6	<.0001
25 to 30	1362	2.7±0.06	42*	7.8±0.6	<.0001
≥ 30	1315	2.7±0.07	75*	7.9±0.4	<.0001
Race/ethnicity					
Non-Hispanic White	2115	2.4±0.05	80*	8.3±0.4	<.0001
Non-Hispanic Black	1387	2.3±0.06	46*	7.4±0.5	<.0001
Others	938	2.8±0.08	26*	7.9±0.7	<.0001
Income (Annual Family Income)					
≤ $24,999	1243	2.8±0.07	47*	8.6±0.5	<.0001
$25,000 to $ $54,999	3197	2.5±0.04	56*	7.9±0.4	<.0001
$55,000 to $74,999	882	2.4±0.07	22*	7.7±0.2	<.0001
≥ $74,999	892	2.7±0.08	27*	7.1±0.8	<.0001
Education (N, %)					
≤ 12th grade	2441	2.5±0.05	78*	8.0±0.4	<.0001
> 12th grade	1999	2.6±0.05	74*	7.9±0.4	<.0001
Alcohol use (N, %)					
yes	3050	2.7±0.04	115*	7.9±0.3	<.0001
No	1390	2.2±0.06	37*	8.2±0.7	<.0001
Ever smoked (N, %)					
yes	2218	2.6±0.05	99*	8.6±0.4	<.0001
No	2222	2.4±0.05	53*	6.8±0.4	<.0001
US Birth (N, %)					
yes	3307	2.6±0.04	125*	8.3±0.3	<.0001
No	1133	2.4±0.07	27*	6.3±0.6	<.0001
Work activity (N, %)					
yes	2025	2.4±0.05	19*	7.5±0.9	<.0001
No	2415	2.6±0.05	133*	8.0±0.3	<.0001
Eat frozen food (N, %)					
yes	1624	2.6±0.06	131*	8.1±0.3	<.0001
No	2816	2.5±0.04	21*	7.2±0.8	<.0001
Liver/kidney diseases (N, %)					
yes	100	2.6±0.3	11*	8.0±0.9	<.0001
No	4340	2.5±0.04	141*	7.9±0.3	<.0001

Table S2: Analysis of significantly levels of Benzophenone-3 (ng/ml) associated with the PCa cases and non-cases; NHANES 2005-2015 (* P < 0.05).

**Variables**	**Non-Cases (N=4440)**		**PCa cases (N=152)**		
	**n**	**Mean ± se**	**n**	**Mean ± se**	
Age					**P-values**
20 – 49	2195	5.5±1.0	9*	15.0±0.7	<.0001
50 – 69	1481	9.0±0.2	61*	18.7±0.3	<.0001
≥ 70	764	8.3±0.3	82*	23.7±0.3	<.0001
BMI					
≤ 25	1763	7.4±0.16	35*	22.3±0.6	<.0001
25 to 30	1362	6.7±0.17	42*	21.3±0.5	<.0001
≥ 30	1315	7.1±0.17	75*	20.7±0.4	<.0001
Race/ethnicity					
Non-Hispanic White	2115	7.3±0.14	80*	21.6±0.4	<.0001
Non-Hispanic Black	1387	6.8±0.18	46*	20.7±0.6	<.0001
Others	938	6.4±0.18	26*	21.0±0.7	<.0001
Income (Annual Family Income)					
≤ $24,999	1243	6.7±0.17	47*	21.7±0.5	<.0001
$25,000 to $ $54,999	3197	6.8±0.18	56*	21.4±0.5	<.0001
$55,000 to $74,999	882	6.6±0.20	22*	21.3±0.9	<.0001
≥ $74,999	892	6.4±0.16	27*	20.2±0.8	<.0001
Education (N, %)					
≤ 12th grade	2441	7.3±0.13	78*	20.9±0.4	<.0001
> 12th grade	1999	6.6±0.13	74*	21.6±0.4	<.0001
Alcohol use (N, %)					
yes	3050	6.8±0.11	115*	21.1±0.3	<.0001
No	1390	6.9±0.12	37*	21.7±0.5	<.0001
Ever smoked (N, %)					
yes	2218	6.7±0.13	99*	21.6±0.4	<.0001
No	2222	6.5±0.12	53*	20.1±0.5	<.0001
US Birth (N, %)					
yes	3307	7.3±0.11	125*	21.6±0.3	<.0001
No	1133	6.7±0.19	27*	19.4±0.7	<.0001
Work activity (N, %)					
yes	2025	6.8±0.13	19*	20.7±0.7	<.0001
No	2415	7.4±0.13	133	21.3±0.3	<.0001
Eat frozen food (N, %)					
yes	1624	7.1±0.6	131*	21.4±0.3	<.0001
No	2816	7.2±0.12	21*	20.0±0.8	<.0001
Liver/kidney diseases (N, %)					
yes	100	7.4±0.61	11*	22.0±1.0	<.0001
No	4340	7.1±0.09	141*	21.2±0.3	<.0001

Table S3: Analysis of significant levels of Triclosan (ng/ml) associated with the PCa cases and non-cases; NHANES 2005-2015 (* P < 0.05).

Variables	Non-Cases (N=4440)		PCa cases (N=152)		
	n	Mean ± se	n	Mean ± se	
Age					P-values
20 – 49	2195	5.6±0.09	9*	10.7±0.3	<.0001
50 – 69	1481	6.2±0.11	61*	14.8±0.2	<.0001
≥ 70	764	6.1±0.16	82*	19.3±0.3	<.0001
BMI					
≤ 25	1763	6.4±0.11	35*	17.2±0.5	<.0001
25 to 30	1362	5.6±0.11	42*	16.6±0.5	<.0001
≥ 30	1315	5.7±0.11	75*	17.1±0.4	<.0001
Race/ethnicity					
Non-Hispanic White	2115	6.4±0.16	80*	17.1±0.4	<.0001
Non-Hispanic Black	1387	6.1±0.11	46*	16.6±0.5	<.0001
Others	938	5.8±0.15	26*	17.4±0.6	<.0001
Income (Annual Family Income)					
≤ $24,999	1243	6.3±0.16	47*	17.7±0.4	<.0001
$25,000 to $ $54,999	3197	6.1±0.16	56*	16.7±0.5	<.0001
$55,000 to $74,999	882	5.9±0.19	22*	16.8±0.9	<.0001
≥ $74,999	892	6.1±0.18	27*	16.5±0.6	<.0001
Education (N, %)					
≤ 12th grade	2441	6.1±0.15	78*	17.0±0.4	<.0001
> 12th grade	1999	6.3±0.16	74*	17.1±0.4	<.0001
Alcohol use (N, %)					
yes	3050	5.9±0.11	115*	17.0±0.3	<.0001
No	1390	6.3±0.12	37*	17.3±0.5	<.0001
Ever smoked (N, %)					
yes	2218	6.1±0.15	99*	17.4±0.3	<.0001
No	2222	6.2±0.19	53*	20.1±0.5	<.0001
US Birth (N, %)					
yes	3307	6.3±0.14	125*	17.3±0.3	<.0001
No	1133	6.1±0.18	27*	16.3±0.7	<.0001
Work activity (N, %)					
yes	2025	5.8±0.14	19*	16.5±0.6	<.0001
No	2415	6.2±0.12	133*	17.1±0.3	<.0001
Eat frozen food (N, %)					
yes	1624	6.5±0.4	131*	17.1±0.3	<.0001
No	2816	5.9±1.1	21*	16.3±0.8	<.0001
Liver/kidney diseases (N, %)					
yes	100	6.3±.016	11*	17.4±1.0	<.0001
No	4340	6.2±0.12	141*	17.0±0.3	<.0001

Table S4: Analysis of significant levels of Ethylparaben (ng/ml) associated with the PCa cases and non-cases; NHANES 2005-2015 (* P < 0.05)

**Variables**	**Non-Cases (N=4440)**		**PCa cases (N=152)**		
	**n**	**Mean ± se**	**n**	**Mean ± se**	
Age					**P-values**
20 – 49	2195	2.2±0.06	9*	9.9±0.8	<.0001
50 – 69	1481	2.4±0.08	61*	10.0±0.4	<.0001
≥ 70	764	2.4±0.11	82*	15.1±0.3	<.0001
BMI					
≤ 25	1763	2.4±0.07	35*	13.3±0.7	<.0001
25 to 30	1362	2.1±0.01	42*	12.0±0.7	<.0001
≥ 30	1315	2.3±0.08	75*	12.9±0.4	<.0001
Race/ethnicity					
Non-Hispanic White	2115	2.1±0.05	80*	12.4±0.5	<.0001
Non-Hispanic Black	1387	2.3±0.06	46*	12.8±0.6	<.0001
Others	938	2.2±0.10	26*	13.7±0.8	<.0001
Income (Annual Family Income)					
≤ $24,999	1243	2.4±0.06	47*	13.3±0.5	<.0001
$25,000 to $ $54,999	3197	2.0±0.06	56*	12.4±0.6	<.0001
$55,000 to $74,999	882	2.1±0.08	22*	12.8±0.9	<.0001
≥ $74,999	892	2.2±0.07	27*	12.3±0.8	<.0001
Education (N, %)					
≤ 12th grade	2441	2.0±0.06	78*	12.3±0.5	<.0001
> 12th grade	1999	2.1±0.08	74*	13.2±0.5	<.0001
Alcohol use (N, %)					
yes	3050	2.3±0.06	115*	12.5±0.4	<.0001
No	1390	2.4±0.10	37*	13.6±0.6	<.0001
Ever smoked (N, %)					
yes	2218	2.4±0.06	99*	13.0±0.4	<.0001
No	2222	2.0±0.06	53*	12.3±0.5	<.0001
US Birth (N, %)					
yes	3307	2.3±0.06	125*	13.0±0.4	<.0001
No	1133	2.2±0.10	27*	11.6±0.7	<.0001
Work activity (N, %)					
yes	2025	2.2±0.06	19*	12.3±0.9	<.0001
No	2415	2.4±0.08	133*	12.8±0.4	0.1475
Eat frozen food (N, %)					
yes	1624	2.4±0.06	131*	12.9±0.4	<.0001
No	2816	2.3±0.04	21*	12.0±0.9	<.0001
Liver/kidney diseases (N, %)					
yes	100	2.4±0.07	11*	13.8±1.2	<.0001
No	4340	2.1±0.05	141*	12.7±0.3	<.0001

Table S5: Analysis of significant levels of Methylparaben (ng/ml) associated with the PCa cases and non-cases; NHANES 2005-2015 (* P < 0.05).

Variables	Non-Cases (N=4440)		PCa cases (N=152)		
	**n**	**Mean ± se**	**n**	**Mean ± se**	
Age					**P-values**
20 – 49	2195	8.8±0.09	9**	9.3±0.3	0.7202
50 – 69	1481	9.9±0.1	61*	14.6±0.3	<.0001
≥ 70	764	9.8±0.2	82*	18.9±0.3	<.0001
BMI					
≤ 25	1763	9.4±0.09	35	17.0±0.6	<.0001
25 to 30	1362	9.3±0.10	42	16.0±0.7	<.0001
≥ 30	1315	9.3±0.10	75	17.0±0.4	<.0001
Race/ethnicity					
Non-Hispanic White	2115	9.1±0.07	80	16.5±0.4	<.0001
Non-Hispanic Black	1387	9.3±0.09	46	16.3±0.6	<.0001
Others	938	9.6±0.10	26	17.3±0.8	<.0001
Income (Annual Family Income)					
≤ $24,999	1243	9.1±1.0	47	17.3±0.6	<.0001
$25,000 to $ $54,999	3197	9.0±0.9	56	16.1±0.5	<.0001
$55,000 to $74,999	882	8.9±0.8	22	17.1±0.9	<.0001
≥ $74,999	892	9.2±0.9	27	16.2±0.7	<.0001
Education (N, %)					
≤ 12th grade	2441	8.8±0.09	78	16.4±0.4	<.0001
> 12th grade	1999	9.9±0.1	74	17.0±0.4	<.0001
Alcohol use (N, %)					
yes	3050	9.5±0.09	115	16.4±0.3	<.0001
No	1390	9.4±0.1	37	17.3±0.6	<.0001
Ever smoked (N, %)					
yes	2218	9.1±0.08	99	17.0±0.4	0.0001
No	2222	9.5±0.09	53	16.0±0.5	<.0001
US Birth (N, %)					
yes	3307	9.4±0.1	125	17.0±0.3	<.0001
No	1133	9.6±0.08	27	15.0±0.7	<.0001
Work activity (N, %)					
yes	2025	9.1±0.08	19	16.1±0.7	<.0001
No	2415	9.5±0.09	133	16.7±0.3	<.0001
Eat frozen food (N, %)					
yes	1624	9.4±0.1	131	17.0±0.3	<.0001
No	2816	9.6±0.08	21	16.±1.0	<.0001
Liver/kidney diseases (N, %)					
yes	100	9.3±0.08	11	17.3±1.2	<.0001
No	4340	9.5±0.09	141	16.5±0.3	<.0001

Table S6: Analysis of significant levels of Propylparaben (ng/ml) and associated with the PCa cases and non-cases; NHANES 2005-2015 (* P < 0.05).

Variables	Non-Cases (N=4440)		PCa cases (N=152)		
	n	Mean ± se	n	Mean ± se	
**Age**					**P-values**
20 – 49	2195	3.1±0.07	9	10.4±0.3	<.0001
50 – 69	1481	4.1±0.10	61	13.8±0.3	<.0001
≥ 70	764	4.3±0.20	82	17.6±0.2	<.0001
BMI					
≤ 25	1763	3.5±0.08	35	16.6±0.9	<.0001
25 to 30	1362	3.6±0.08	42	15.7±0.4	<.0001
≥ 30	1315	3.7±0.07	75	15.3±0.4	<.0001
Race/ethnicity					
Non-Hispanic White	2115	3.3±0.06	80	15.6±0.3	<.0001
Non-Hispanic Black	1387	3.5±0.08	46	15.6±0.5	<.0001
Others	938	3.6±0.07	26	16.1±0.6	<.0001
Income (Annual Family Income)					
≤ $24,999	1243	3.4±0.06	47	16.2±0.4	<.0001
$25,000 to $ $54,999	3197	3.5±0.08	56	15.8±0.4	<.0001
$55,000 to $74,999	882	3.6±0.10	22	15.5±0.7	<.0001
≥ $74,999	892	3.1±0.07	27	15.0±0.7	<.0001
Education (N, %)					
≤ 12th grade	2441	3.7±0.07	78	15.5±0.4	<.0001
> 12th grade	1999	4.1±0.10	74	16.0±0.3	<.0001
Alcohol use (N, %)					
yes	3050	3.6±0.10	115	15.6±0.3	<.0001
No	1390	3.1±0.07	37	16.4±0.6	<.0001
Ever smoked (N, %)					
yes	2218	3.5±0.08	99	15.9±0.3	<.0001
No	2222	3.6±0.10	53	15.4±0.4	<.0001
US Birth (N, %)					
yes	3307	3.6±0.10	125	15.0±0.3	<.0001
No	1133	4.1±0.07	27	14.6±0.6	<.0001
Work activity (N, %)					
yes	2025	3.4±0.06	19	15.7±0.6	<.0001
No	2415	3.5±0.08	133	16.7±0.3	<.0001
Eat frozen food (N, %)					
yes	1624	3.6±0.10	131	15.9±0.3	<.0001
No	2816	3.1±0.07	21	14.8±0.8	<.0001
Liver/kidney diseases (N, %)					
yes	100	3.6±0.10	11	16.1±1.2	<.0001
No	4340	3.2±0.07	141	15.7±0.3	<.0001

Table S7: A summary of genes identified by various gene analysis tools for 81 Overlapping Genes associated with PCa, EPs, and PBs.

#	Term	Gene identified by	Count	Genes
1	Prostate Cancer	DAVID- GAD[1]	35	CDKN1A, CDKN1B, LHB, SERPINE1, KLK3, KLK2, NR3C1, CYP19A1, CYP17A1, CASP9, CCND1, PLAU, CDH1, MYC, DNMT3B, CYP1B1, CD14, NCOA2, NOS2, EGF, UGT2B15, IGF1, ESR1, ESR2, GNMT, VEGFA, AR, IL1B, SELENOP, CYP1A1, BCL2, ID3, PPARA, SHBG, TP53.
2	Prostate cancer	KEGG- PCa Pathway	13	BCL2, AR, CASP9, CCND1, CDKN1A, CDKN1B, EGF, GSK3B, HSP90B1, IGF1, KLK3, MAPK3, TP53.
3	Genes important in the PPI-network	CytoHubba-MCC[2]	10	CCND1, VEGFA, EGF, MYC, CASP3, IGF1, STAT3, TP53, ESR1, CDH1.
4	Hub-Genes1	MCODE- Module1	26	MYC, ESR2, MET, SERPINE1, SOX2, AR, VEGFA, CDKN1A, CCND1, TP53, MAPK3, EGF, ESR1, EGR1, CASP9, HMOX1, IGF, STAT3, CDKN15, CDH1, IL1B, GSK3B, PPARA, MAPK8, EGR1, CASP3.
5	Hub-Genes2	MCODE- Module2	5	BUB1B, TOP2A, UBE2C, RRM2, CENPF.

[1]DAVID- GAD: DAVID-Gene Association Diseases Database (GAD), [2]Maximal Clique Centrality (MCC)

Table S8: Gene ontology and pathway enrichment analysis of EPs and PBs influenced 81 overlapping Genes' and their curated function in PCa (top five in biological process (BP), cell component (CC), molecular function (MF), and in KEGG pathway).

	BP	Term	Count	P-Value	Genes
1	GO:0032355	Response to estradiol	14	$2.76X10^{-16}$	FOXA1, CDKN1B, PCNA, STAT3, ESR1, CASP9, COL1A1, CCND1, MYC, CASP3, ALDH1A2, RARA, DNMT3B, CD38
2	GO:0045944	Positive regulation of transcription from RNA polymerase II promoter	27	$1.63X10^{-13}$	FOXA1, TOP2A, GSK3B, NR1I2, SERPINE1, TWIST1, NR3C1, SOX2, EPCAM, MYC, MAPK3, SREBF1, NCOA2, EGR1, PRRX1, STAT3, IGF1, ESR1, VEGFA, AR, IL1B, RARA, CDH13, PPARA, MET, TP53, ATF3
3	GO:0042493	Response to drug	16	$9.91X10^{-12}$	SREBF1, ABCC4, CDKN1A, CDKN1B, STAT3, COL1A1, CENPF, CCND1, CDH1, MYC, CASP3, CYP1A1, BCL2, DNMT3B, CD38, B2M
4	GO:0045893	Positive regulation of transcription, DNA-templated	18	$1.99X10^{-10}$	EGR1, EGF, STAT3, NR1I2, IGF1, ESR1, ESR2, SOX2, COL1A1, AR, CDH1, MYC, IL1B, RARA, CD38, PPARA, TP53, MAPK3
5	GO:0043066	negative regulation of apoptotic process	17	$2.92X10^{-10}$	GSK3B, CDKN1A, CDKN1B, STAT3, TWIST1, IGF1, HSP90B1, VEGFA, MAPK8, EPCAM, MYC, CASP3, BCL2, RARA, BIRC5, CD38, TP53
	CC	Term	Count	P-Value	
1	GO:0043234	Protein complex	12	$1.78X10^{-06}$	TOP2A, ACTA2, SREBF1, AR, CDKN1A, CDKN1B, MYC, KLK3, NR3C1, TP53, CLU, MAPK3
2	GO:0005654	Nucleoplasm	30	$3.25X10^{-06}$	TOP2A, CDKN1A, CDKN1B, PCNA, NR1I2, JADE2, NR3C1, SOX2, MAPK8, CCND1, MYC, CASP3, DNMT3B, MAPK3, SREBF1, NCOA2, EGR1, RRM2, UBE2C, STAT3, ESR1, ESR2, AR, CENPF, RARA, ID3, BIRC5, PPARA, TP53, ATF3
3	GO:0005615	Extracellular space	20	$4.22X10^{-06}$	EGF, LHB, SERPINE1, KLK3, FASLG, IGF1, CLU, VEGFA, ACTA2, COL1A1, ERBB3, PLAU, IL1B, SELENOP, TNFSF10, CDH13, HMOX1, CD14, MUC4, B2M
4	GO:0005634	Nucleus	44	$5.89X10^{-06}$	FOXA1, TOP2A, GSK3B, CDKN1A, CDKN1B, PCNA, NR1I2, TWIST1, KLK3, FASLG, NR3C1, CLU, HSP90B1, SOX2, CASP9, MAPK8, CCND1, MYC, CASP3, DNMT3B, HMOX1, CD38, MAPK3, SREBF1, NCOA2, EGR1, RRM2, PDHA1, PRRX1, NOS2, STAT3, ESR1, ESR2, AR, CENPF, BCL2, RARA, BAX, SP5, ID3, BIRC5, PPARA, TP53, ATF3
5	GO:0005829	Cytosol	32	$1.24X10^{-05}$	GSK3B, CDKN1A, CDKN1B, BUB1B, CLU, HSP90B1, SOX2, CASP9, MAPK8, CCND1, SULT1E1, MYC, CASP3, STMN1, HMOX1, MAPK3, SREBF1, RRM2, NOS2, UBE2C, IDH1, STAT3, GNMT, ACTA2, AR, CENPF, ALDH1A2, IL1B, BCL2, BAX, BIRC5, TP53
	MF	Term	Count	P-Value	
1	GO:0008134	Transcription factor binding	14	$7.58X10^{-10}$	FOXA1, NCOA2, STAT3, TWIST1, ESR1, AR, CENPF, CCND1, MYC, BCL2, RARA, ID3, PPARA, TP53
2	GO:0043565	Sequence-specific DNA binding	15	$1.30X10^{-07}$	FOXA1, EGR1, PRRX1, NR1I2, NR3C1, ESR1, ESR2, SOX2, AR, MYC, BCL2, RARA, PPARA, TP53, ATF3

3	GO:0005496	Steroid binding	6	$1.57X10^{-07}$	AR, SULT1E1, NR3C1, SHBG, ESR1, ESR2
4	GO:0003707	Steroid hormone receptor activity	7	$2.52X10^{-07}$	AR, NR1I2, RARA, PPARA, NR3C1, ESR1, ESR2
5	GO:0019899	Enzyme binding	12	$4.65X10^{-07}$	TOP2A, AR, MAPK8, PCNA, CCND1, CYP1A1, RARA, HMOX1, BIRC5, ESR1, TP53, ESR2
	KEGG	**Term**	**Count**	**P-Value**	
1	hsa05200:	Pathways in cancer	25	$7.37X10^{-14}$	GSK3B, CDKN1A, CDKN1B, KLK3, FASLG, HSP90B1, CASP9, MAPK8, CCND1, CDH1, MYC, CASP3, MAPK3, NOS2, EGF, STAT3, IGF1, VEGFA, AR, BCL2, RARA, BAX, BIRC5, MET, TP53
2	hsa05215:	Prostate cancer	13	$3.01X10^{-11}$	GSK3B, CDKN1A, CDKN1B, EGF, KLK3, IGF1, HSP90B1, CASP9, AR, CCND1, BCL2, TP53, MAPK3
3	hsa05161:	Hepatitis B	15	$6.83X10^{-11}$	CDKN1A, CDKN1B, PCNA, STAT3, FASLG, CASP9, MAPK8, CCND1, MYC, CASP3, BCL2, BAX, BIRC5, TP53, MAPK3
4	hsa05210:	Colorectal cancer	11	$2.77X10^{-10}$	CASP9, GSK3B, MAPK8, CCND1, MYC, CASP3, BCL2, BAX, BIRC5, TP53, MAPK3
5	hsa04066:	HIF-1 signaling pathway	12	$1.47X10^{-09}$	CDKN1A, CDKN1B, PDHA1, NOS2, EGF, STAT3, SERPINE1, BCL2, HMOX1, IGF1, MAPK3, VEGFA

Table S9: The biological functions of five Hub genes the are closely related in the progression of PCa.

#	Symbol	Full name	Function
1	BUB1B	BUB1 mitotic checkpoint serine/threonine kinase B	Is a critical mitotic checkpoint kinase. Is identified as the top-scoring kinase by RNA interaction. Has a possibility to be a novel antimitotic target and impaired spindle checkpoint function in any form of cancer, including PCa [55,94].
2	TOP2A	DNA Topoisomerase II alpha	Is a critical nuclear enzyme for chromosome condensation, DNA mitosis, and cell division. May be utilized as a biomarker that indicates poor prognosis and may function as a treatment target for PCa. Has been suggested to directly interact with P53, a well-known tumor suppressor protein. Regulates the topology structure of DNA and cell cycle progression [50,51].
3	UBE2C	Ubiquitin-conjugating enzyme complex E2 C	Plays as an independent prognostic factor of PCa, and recreated a critical role in the pathway of PCa (WNT-β-catenin signaling pathway and NOTCH signaling pathway) [51].
4	RRM2	Ribonucleotide reductase regulatory subunit M2	Well-known as a functional catalytic site in controlling the cell cycle by regulating DNA repair and replication. An enzyme that determines the rate of DNA synthesis and repair. It was considered to be a biomarker to indicate recurrence in PCa patients with low risk [50,51].
5	CENPF	Centromere protein F	Functions in the centromere-kinetochore complex and chromosomal segregation and the upregulation of CENPF are connected to aggressive PCa [53,54].

Table S10: The variables that were inputted as categorical Covariates

#	Variables	Value Description	Detailed information
1	Age	20 - 49	Obtained from demographic data, and classified into three groups
		50 - 69	
		≥ 70	
2	BMI	< 25	Obtained from body measures data, and classified into three groups
		25 to 30	
		>30	
3	Race	Non-Hispanic White	Obtained from demographic data, and classified into three groups
		Non-Hispanic Black	
		Other Race	
4	Income	≤ $24,999	Obtained from demographic data, and classified into four groups
		$25,000 to $ $54,999	
		$55,000 to $74,999	
		≥ $74,999	
5	Education level	≤ 12th grade	Obtained from demographic data, and classified into two groups
		> 12th grade	
6	US Birth	Yes	In what country were you born? U.S. born answer "yes or no"
		No	
7	Physical activity	Yes	Do you participate in a vigorous-intensity exercise which causes significant increases in breathing or heart rate (<10 min) continuously? "Yes or no"-did not they ask how often on a week or so?
		No	
8	Eat frozen food	Yes	During the past 30 days, how often did you eat frozen meals? "Yes or no"
		No	
9	Alcohol use	Yes	In any one year, have you had at least 12 drinks of any type of alcoholic beverage? "Yes or no"
		No	
10	Smoking	Yes	Smoking status ["Have you smoked at least 100 cigarettes in your entire life? "Yes or no"
		No	
11	Liver diseases	Yes	Were you diagnosed with liver diseases? "Yes or no"
		No	
12	Kidney disease	Yes	Were you diagnosed with liver diseases? "Yes or no"
		No	

Table S11: The variables that were inputted as numerical Covariates

#	Variables*	Value Description
1	AGE	Years
2	WT	Kg
3	LBXTC	mg/dl
4	LDL	mg/dl
5	HDL	mg/dl
6	TRIGLY	mg/dl

AGE: Age, WT: Weight, BXTC: total cholesterol, LDL: low-density lipoprotein (LDL), HDL: high-density lipoprotein, and TRIGLY: triglycerides (TRIGLY).
